# Monocyte-derived dendritic cells promote T follicular helper cell differentiation

**DOI:** 10.1002/emmm.201403841

**Published:** 2014-04-11

**Authors:** Svetoslav Chakarov, Nicolas Fazilleau

**Affiliations:** 1 Centre de Physiopathologie de Toulouse PurpanToulouse, France; 2 INSERM, U1043Toulouse, France; 3 CNRS, UMR5282Toulouse, France; 4 Université Toulouse III Paul-SabatierToulouse, France

**Keywords:** adjuvant, antibody, dendritic cell, T lymphocyte, Toll-like receptor

## Abstract

To be effective, protein priming must induce the development of a distinct lineage of CD4^+^ T cells named T follicular helper (Tfh) cells, which regulate the differentiation of high-affinity memory B cells and long-lived plasma cells. In this context, we tested how adjuvantation with CpG, the Toll-like receptor 9 agonist used in clinics, contributes to antigen-specific T-cell-dependent B-cell responses *in vivo*. We found that addition of CpG to other vaccine adjuvant increased the differentiation of antigen-specific Tfh cells without changing the overall magnitude of the T-cell response. This phenomenon correlated with an enhancement of the germinal centre reaction, antigen-specific plasma cells and circulating antibodies. We comprehensively demonstrated that, in addition to the classical Tfh-cell differentiation mediated by conventional DC, the promoting effect due to CpG was orchestrated *in vivo* by antigen presentation and IL-6 secreted by monocyte-derived dendritic cells (DC) as shown in their absence. Thus, while conventional DC initiate T-cell responses, targeting monocyte-derived DC specifically enhances the Tfh programme needed to regulate high-affinity B-cell protection *in vivo*.

## Introduction

Most vaccines are designed empirically using attenuated pathogens as the source of foreign antigens (Ag). Improving vaccine efficacy has, however, proven difficult, mainly because the fundamental immune mechanisms of vaccine action remain elusive. Toll-like receptors (TLR) are innate immune receptors that specifically recognise pathogen challenges and are pivotal for initiating inflammation but also for the priming of adaptive immune responses (Banchereau & Steinman, 1998; Janeway & Medzhitov, 2002; Beutler *et al*, 2006). TLR ligands are thus widely used as vaccine adjuvants in different clinical settings (Klinman, 2004; Pulendran & Ahmed, 2011). Among them, CpG oligonucleotides (ODN) are recognised by TLR9, a sensor for hypomethylated CpG sequences such as found in DNA of bacterial or viral origin, and is localised to the endosomal membrane of B cells and dendritic cells (DC) (Klinman, 2004). Upon recognition of CpG, the intracellular adapter molecule myeloid differentiation factor 88 (MyD88) interacts and activates interferon regulatory factor 7 (IRF-7), which is required for transcriptional activity of type I interferon (IFN). MyD88 engagement also leads to the activation of nuclear factor-κB (NF-κB), which in turn induces the transcription of pro-inflammatory cytokines such as IL-6 and TNF-α.

Three distinct classes of synthetic CpG ODN exist based on their sequence and structure (phosphorothioate or mixed phosphodiester-phosphorothioate backbones): CpG-A, CpG-B and CpG-C (Krieg, 2002; Klinman, 2004). Depending on the type of CpG, the retention time in endosomes and the nature of endosomal compartments vary and result in either NF-κB or IRF-7 activation (Honda *et al*, 2005; Engel & Barton, 2010). Using a mouse model that lacks MyD88 selectively in DC or in B cells, soluble CpG-B has been shown to augment the T-cell-dependent antibody (Ab) response to protein selectively through DC (Hou *et al*, 2011). In contrast, the adjuvant activity of CpG-B on Ab response is dependent on MyD88 expression in B cells when CpG-B is delivered in virus-like particle (Hou *et al*, 2011) and CpG-B was shown to directly stimulate B cells to undergo isotype switching to IgG2a (Jegerlehner *et al*, 2007).

Protein vaccines promote long-term immunity through the differentiation of B-cell responses that are controlled in secondary lymphoid tissues by a distinct lineage of T-helper cells, named T follicular helper (Tfh) cells, through a combination of specific TCR-peptide-MHCII (pMHCII) interactions, engagement of co-stimulatory molecules and cytokine delivery (Fazilleau *et al*, 2009a; Crotty, 2011). It has been shown that IL-6, IL-21 and the strength of TCR binding promote *in vivo* Tfh-cell development that occurs preferentially in lymphoid organs draining the site of immunisation (Fazilleau *et al*, 2007, 2009b; Suto *et al*, 2008; Vogelzang *et al*, 2008). Recent studies have demonstrated that Bcl-6 is the master transcriptional regulator of Tfh-cell differentiation (Johnston *et al*, 2009; Nurieva *et al*, 2009; Yu *et al*, 2009). Among others, Bcl-6 induces the expression of the chemokine receptor CXCR5, which promotes Tfh-cell migration in CXCL13-rich areas where they regulate the outcome of the B-cell response (Forster *et al*, 1996; Fazilleau *et al*, 2009a). Additionally, while Tfh cells control B-cell maturation, interactions with B cells are reciprocally essential for Tfh-cell differentiation as shown with *in vivo* impairment of Tfh-cell development in the absence of T-B cooperation (Johnston *et al*, 2009; Nurieva *et al*, 2009).

Adjuvant combinations result in synergistic enhancement of the immune response. Notably, adjuvantation with CpG-B was shown to markedly increase Tfh- and B-cell neonatal responses (Mastelic *et al*, 2012). Moreover, CpG-B adjuvantation of aluminium-containing adjuvant (Alum) or of MF59, a squalene-based adjuvant used in clinical settings, induces more potent Ab response (Wack *et al*, 2008). However, the molecular and cellular mechanisms responsible for this increase in T-cell-dependent B-cell responses after the addition of CpG-B remained unknown. Here, using three Ag models for which we have direct access in wild-type (WT) mice to antigen (Ag)-specific T cells, Ag-specific B cells and Ag-presenting DC, we document that the adjuvantation with CpG-B of other vaccine adjuvant enhances Ag-specific Tfh cells without changing the overall extent of the Ag-specific T-cell pool. Consequently, it enhances Ag-specific memory B-cell development and Ab response. Furthermore, we show that Ag-presenting monocyte-derived DC (moDC) are responsible for the increase in Tfh cells as shown *in vivo* in their absence. We demonstrate that moDC mediate this phenomenon through the secretion of IL-6. Overall, these results suggest that targeting moDC can imprint the specialised programme of effector Tfh function needed to promote high-affinity B-cell immunity *in vivo* that, ultimately, boosts protein vaccine efficacy.

## Results

### Adjuvantation with CpG-B promotes Ag-specific Tfh-cell development

**Figure 1:**
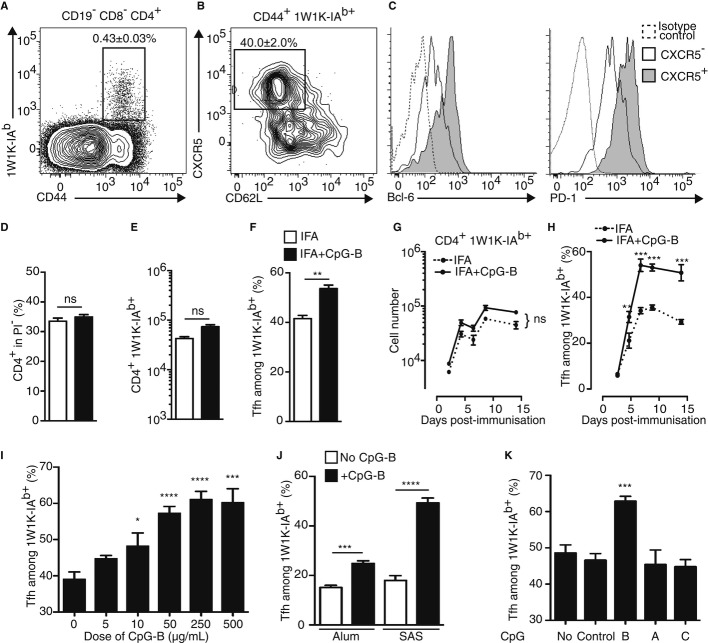
Adjuvantation with CpG-B of other vaccine adjuvant promotes Ag-specific Tfh-cell development. A–C Nine days after s.c. immunisation with 40 μg of 1W1K in IFA, dLN were analysed for the detection of 1W1K-specific activated CD4^+^ T cells (1W1K-IA^b+^CD44^+^) (A) and 1W1K-specific Tfh cells (CXCR5^+^CD62L^−^) (B) as shown by Bcl-6 and PD-1 expression (C) (*n* = 5/condition, mean ± s.e.m.). D–F Frequency of CD4^+^ T cells (D), 1W1K-specific activated CD4^+^ T cells (E) and frequency of Tfh in 1W1K-specific CD4^+^ T cells (F) 9 days after s.c. immunisation with 40 μg of 1W1K in IFA or IFA+CpG-B (*n* = 5/group, mean ± s.e.m.). G, H Number of total 1W1K-specific CD4^+^ T cells (G) and frequency of 1W1K-specific Tfh (H) in dLN after s.c. immunisation with 1W1K in IFA (open circle) or IFA+CpG-B (solid circle) (*n* = 5/condition, mean ± s.e.m.). I–K Frequency of Tfh cells among 1W1K-specific CD4^+^ T cells in dLN 9 days after s.c. immunisation with IFA mixed with different dose of CpG-B (I), with other adjuvant (Alum, SAS) mixed with CpG-B (J) or with IFA mixed with different type of CpG (K) (*n* = 5/condition, mean ± s.e.m.). Data information: Data are representative of at least four independent experiments. ns, nonsignificant; *P <* *0.05; **P ≤* *0.01; ***P ≤* *0.005; ****P ≤* *0.001.

We first examined the I-A^b^-restricted murine T-cell response to a peptide variant (EAWGALANKAVDKA, called 1W1K peptide hereafter) of the I-E alpha chain immunodominant peptide 52-68 (Ea) (Rudensky *et al*, 1991) in C57Bl/6 wild-type mice. We followed the 1W1K-specific CD4^+^ T cells with the corresponding pMHCII tetramer in the draining LN (dLN) after immunisation with 1W1K in incomplete Freund's adjuvant (IFA). At day 9, the peak of the effector response, we detected the CD44^+^ and pMHCII tetramer^+^ 1W1K-specific CD4^+^ T cells in the dLN (Fig 1A). We further analysed the 1W1K-specific CD4^+^ T cells that were CD62L^lo^ CXCR5^+^ corresponding to Tfh cells (Fig 1B), as confirmed by Bcl-6 and PD-1 expression (Fig 1C). Since CpG-B elevates the total neonatal Tfh-cell pool to the level of adults (Mastelic *et al*, 2012), we tested whether CpG-B adjuvantation of IFA has a similar impact on Ag-specific Tfh cells. We found that addition of CpG-B did not statistically change the number of CD4^+^ T cells in the dLN (Fig 1D) or the amount of 1W1K-specific CD4^+^ T cells at the peak of the effector response (Fig 1E). Strikingly, we observed that addition of CpG-B induced an increase in Tfh cells among the 1W1K-specific CD4^+^ T cells (Fig 1F). Moreover, no variation was found in the kinetics of the 1W1K-specific CD4^+^ T-cell response in both immunising conditions (Fig 1G) and the enhancing effect due to adjuvantation with CpG-B on the Tfh compartment was observed at all time points tested from day 5 after immunisation (Fig 1H).

Interestingly, we observed that this phenomenon was dose dependent and reached a plateau for a dose of 250 μg/ml (Fig 1I). As the adjuvanticity of CpG-B may vary with the physical context in which it is presented to TLR9, we next tested whether CpG-B also enhances Tfh-cell responses when added to two other types of adjuvant or when adjuvantation of IFA is done with other type of CpG. While IFA is a “water-in-oil” adjuvant, Alum is an aqueous adjuvant (Naim *et al*, 1997) and SAS is an “oil-in-water” adjuvant composed of monophosphoryl lipid A and trehalose dicorynomycolate in metabolisable squalene oil (Baldridge & Crane, 1999). We found that CpG-B adjuvantation of Alum and SAS also increased 1W1K-specific Tfh cells (Fig 1J). Moreover, we found that CpG-A, CpG-C and control CpG-B had no detectable effect on the frequency of 1W1K-specific Tfh cells in the dLN 9 days after immunisation compared to IFA alone (Fig 1K).

Together, these results demonstrate that adjuvantation with CpG-B of other vaccine adjuvant enhances the pool of Ag-specific Tfh cells *in vivo* without affecting the overall magnitude and the dynamics of the Ag-specific CD4^+^ T-cell compartment.

### Adjuvantation with CpG-B boosts Ag-specific B-cell response

**Figure 2:**
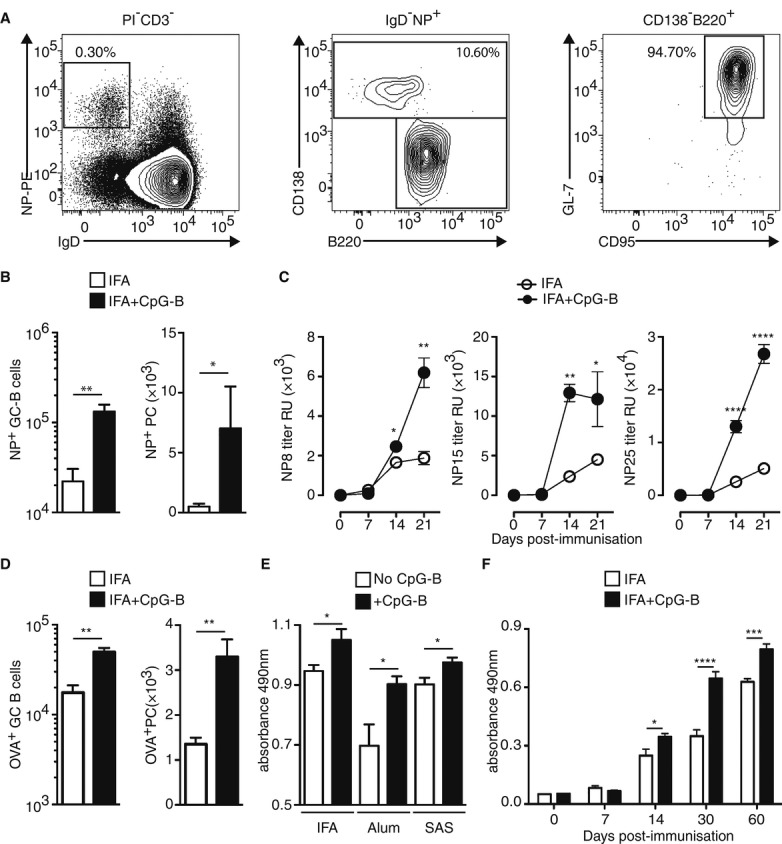
Addition of CpG-B boosts OVA-specific B-cell responses. A Fourteen days after s.c. immunisation with 100 μg of NP-OVA in IFA, dLN and sera were analysed to estimate NP-specific B cells and Ig levels, respectively. Representative example of FACS profiles: NP-specific B cells (left panel), among which plasma cells (PC: CD138^+^; middle panel) and GC-B cells (CD138^−^B220^+^GL-7^+^CD95^+^; right panel). B Numbers of NP-specific GC-B cells and NP-specific PC in dLN after immunisation with IFA or IFA+CpG-B (*n* = 5/group; mean ± s.e.m.). C Levels of NP-specific IgG in sera after s.c. immunisation with NP-OVA in IFA or IFA+CpG-B (*n* = 5/group, mean ± s.e.m.). D 14 days after s.c. immunisation with 100 μg of OVA, numbers of OVA-specific GC-B cells and OVA-specific PC in dLN (*n* = 5/group; mean ± s.e.m.). E, F Levels of OVA-specific IgG in sera of mice 14 days after s.c. immunisation with OVA in adjuvant or adjuvant+CpG-B (*n* = 5/condition, mean ± s.e.m.) (E) and over time after s.c. immunisation using IFA (F). Data information: Data are representative of at least three independent experiments. ns, nonsignificant; *P <* *0.05; **P ≤* *0.01; ***P ≤* *0.005; ****P ≤* *0.001.

The B-cell response to the hapten 4-hydroxy-3-nitrophenylacetyl (NP) in WT animals is a valuable immunisation model that can be monitored via the polyclonal Ig repertoires. NP-specific B cells can be detected by flow cytometry as CD3^−^ IgD^−^ cells that bind specifically to phycoerythrin (PE)-conjugated NP (Fig 2A). Two functionally distinct populations can be examined upon phenotypic analysis: CD138^+^ plasma cells (PC) and CD138^−^ B220^+^ GL-7^+^ CD95^+^ germinal centre (GC)-B cells (Fig 2A) (McHeyzer-Williams & McHeyzer-Williams, 2004). Using this strategy, we observed that the addition of CpG-B increased both the NP-specific GC-B cells and PC (Fig 2B). Moreover, we found a significant increase from day 14 after immunisation in serum NP-specific IgG when IFA is supplemented with CpG-B. This increase was observed irrespective of Ig affinity for the Ag as shown using NP8 (high affinity), NP15 (intermediate and low affinity) and NP25 (all affinity) (Fig 2C). Moreover, using ovalbumin (OVA) as Ag, we can also track OVA-specific B cells by flow cytometry (Supplementary Fig S1). In this context, we also found a significant increase in OVA-specific GC-B cells and PC (Fig 2D) and in OVA-specific IgG circulating in animals immunised with IFA, Alum or SAS supplemented with CpG-B (Fig 2E), showing that our observations were not peculiar to one distinct Ag. Interestingly, we also found that increase in Ag-specific Tfh-cell-dependent B-cell responses after adjuvantation with CpG-B of vaccine formulation could be observed at later time points after immunisation. More precisely, we found an increase in the OVA-specific Ig response (Fig 2F) and the pool of 1W1K-specific Tfh cells (Supplementary Fig S2) 60 days after immunisation. Altogether, these data demonstrate that adjuvantation with CpG-B of other vaccine adjuvant intensifies specifically Ag-specific T-cell-dependent Ab responses *in vivo*.

### DC mediate the increase in T-cell-dependent B-cell response due to CpG-B adjuvantation

**Figure 3:**
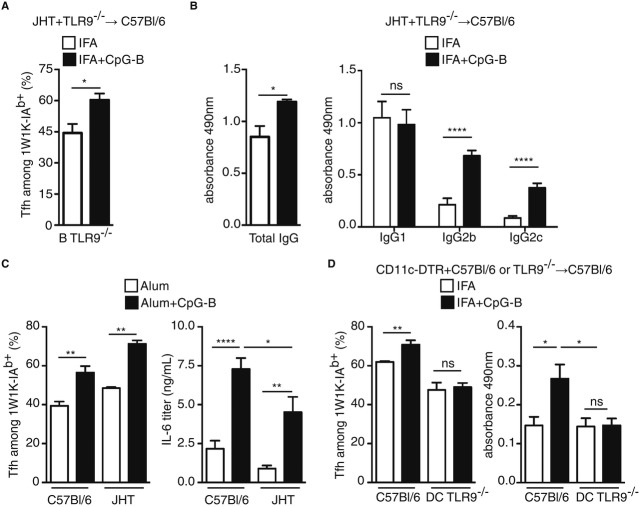
Impact of CpG-B on Tfh-cell differentiation relies on CD11c^+^ cells. A, BEight weeks after reconstitution, chimeric JHT (70%) + TLR9^−/−^ (30%) → C57Bl/6 were s.c. immunised with 1W1K or OVA in IFA or IFA+CpG-B. Frequency of Tfh among 1W1K-specific CD4^+^ T cells in dLN 9 days after s.c. immunisation (*n* = 5/group, mean ± s.e.m.) (A) and levels of OVA-specific IgG, IgG1, IgG2b and IgG2c in the sera of mice 14 days after s.c. immunisation (*n* = 5/condition, mean ± s.e.m.) (B). C Five days after i.p. injection of JHT or C57Bl/6 littermates with 40 μg of 1W1K in Alum or Alum+CpG-B, spleens were collected and analysed. Frequency of Tfh among 1W1K-specific CD4^+^ T cells 9 days after i.p. injection is shown as well as serum concentrations of IL-6 estimated by ELISA 6 h after the i.p. injection (*n* = 5/group mean ± s.e.m.). D Eight weeks after reconstitution, chimeric CD11cDTR (70%) + (30%) C57Bl/6 or TLR9^−/−^ → C57Bl/6 mice were treated every 2 days with DTx and s.c. immunised with 1W1K or OVA in IFA or IFA+CpG-B. Frequency of Tfh among 1W1K-specific CD4^+^ T cells in dLN 9 days after s.c. immunisation (*n* = 5/group, mean ± s.e.m.) and levels of OVA-specific IgG, in the sera of mice 14 days after s.c. immunisation (*n* = 5/group, mean ± s.e.m.). Data information: Data are representative of at least three independent experiments. ns, nonsignificant; *P < 0.05; **P ≤* *0.01; ****P ≤* *0.001.

While Tfh cells control B-cell maturation, interactions with B cells are reciprocally essential for Tfh-cell differentiation (Qi *et al*, 2008; Vogelzang *et al*, 2008; Choi *et al*, 2011). Interestingly, CpG-B was shown earlier to promote Ig class switch recombination in a B-cell intrinsic fashion (Jegerlehner *et al*, 2007). Thus, we next explored whether the increase in Ag-specific Tfh cell and B cells upon CpG-B addition is B cell mediated. First, we found that adjuvantation with CpG-B promotes preferentially the follicular pathway as shown with increased number and size of GC day 21 after immunisation (Supplementary Fig S3). Moreover, a bone marrow (BM) chimeric system in which TLR9 deficiency was restricted to B cells was developed. We took advantage of the JHT mouse model that totally lacks B cells (Gu *et al*, 1993). C57Bl/6 recipients were lethally irradiated before reconstitution with a mix of JHT and TLR9^−/−^ BM. The chimeras were immunised and the 1W1K-specific Tfh-cell response was monitored. We found that absence of TLR9 signalling in B cells had no effect on the enhancing effect of Tfh-cell development due to CpG-B addition (Fig 3A). Similarly, when chimeric animals were immunised with OVA, we showed that the increase in the OVA-specific IgG due to CpG-B addition was also observed even in the absence of TLR9 signalling in B cells (Fig 3B). We then immunised JHT and WT littermates with 1W1K in Alum or Alum with CpG-B and found that CpG-B adjuvantation enhanced 1W1K-specific Tfh-cell differentiation even in the absence of functional B cells (Fig 3C). Of note, we found as expected an increase in serum IL-6 after CpG-B adjuvantation in both C57Bl/6 and JHT littermates (Fig 3C). However, less IL-6 in the serum of immunised animals was observed in CpG-B-sensitised JHT mice showing that B cells, as expected, secrete large amount of IL-6 in response to CpG-B (Fig 3C).

To address whether DC cells drive the effect of CpG-B addition to other adjuvant on T-cell-dependent B-cell response, a BM chimeric system in which TLR9 deficiency was restricted to CD11c^+^ cells was developed. For this purpose, we used a transgenic (TG) mouse model of CD11c-DTR in which CD11c^+^ cells can be depleted after DTx injection (Jung *et al*, 2002). As described above, C57Bl/6 recipients were lethally irradiated before reconstitution with BM from CD11c-DTR and TLR9^−/−^ mice. The resulting chimeras were treated with DTx, which resulted in the specific depletion of CD11c^+^ cells from CD11c-DTR origin, and were immunised with 1W1K. We found that the absence of TLR9 signalling in CD11c^+^ cells resulted in the absence of increase in Tfh-cell differentiation due to CpG-B (Fig 3D). Similarly, when these chimeric animals were treated with DTx and immunised with OVA, we showed that the increase in OVA-specific IgG due to CpG-B addition was observed only in presence of TLR9 signalling in CD11c^+^ cells (Fig 3D). Altogether, these data show that the increase in Tfh-cell development upon the addition of CpG-B to other vaccine adjuvant depends on TLR signalling in CD11c^+^ cells, but not in B cells, even if these latter cells secrete large amount of IL-6 in response to CpG-B.

### CD11b^+^ conventional DC and monocyte-derived DC present the Ag to CD4^+^ T cells in the dLN

**Figure 4:**
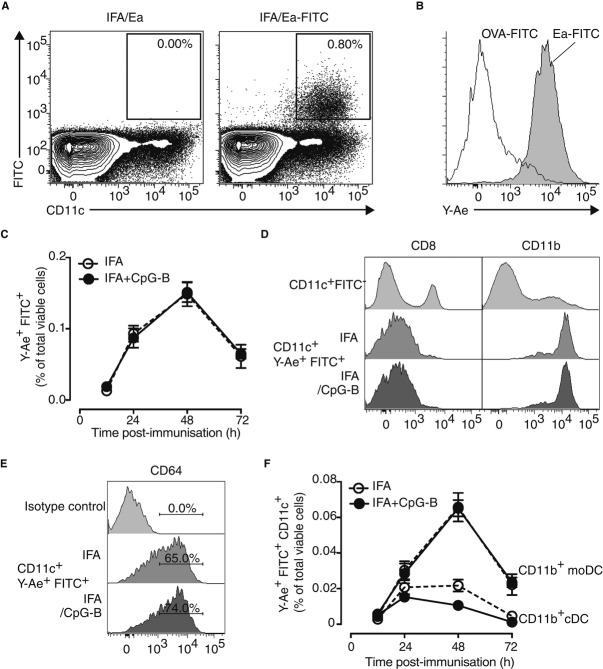
Ag-presenting DC in the dLN are CD11b^+^ conventional and monocyte-derived DC. A DC that captured and present Ag (CD11c^+^FITC^+^Y-Ae^+^) in dLN of mice 2 days after s.c. immunisation with Ea-FITC emulsified in IFA. B Histograms of Y-Ae staining correspond to mice s.c. immunised either with OVA-FITC or Ea-FITC in IFA. C Kinetics of Y-Ae^+^ FITC^+^ CD11c^+^ cells in the dLN after s.c. immunisation after IFA or IFA+CpG-B (IFA, open circle; IFA+CpG-B, solid circle) (*n* = 4/condition, mean ± s.e.m.). D, E Two days after s.c. immunisation with Ea-FITC in IFA or IFA+CpG-B, Y-Ae^+^ FITC^+^CD11c^+^ cells from dLN were analysed for CD8, CD11b expression (D) and for CD64 (E). F Kinetics of conventional DC (cDC, Y-Ae^+^ FITC^+^ CD11c^+^ CD11b^+^ CD64^−^) and monocyte-derived DC (moDC, Y-Ae^+^ FITC^+^ CD11c^+^ CD11b^+^ CD64^+^) in the dLN after s.c. immunisation with IFA or IFA+CpG-B (IFA, open circle; IFA+CpG-B, solid circle) (*n* = 3/group, mean ± s.e.m.). Data information: Data are representative of at least five independent experiments.

To track Ag-presenting DC, we took advantage of two technical approaches allowing us to follow and isolate directly the Ag-presenting DC in WT animals after immunisation. First, to be able to track cells that had captured the Ag, we used fluorescein isothiocyanate (FITC)-conjugated Ag (Fig 4A). Second, we used the Y-Ae monoclonal Ab (mAb) that specifically recognises the pMHCII complex I-A^b^-Ea in C57Bl/6 (Murphy *et al*, 1989, 1992; Itano *et al*, 2003). After immunisation with Ea-FITC, we found that all the CD11c^+^ DC in the dLN that had captured the Ag were Y-Ae^+^ (Fig 4B). Alternatively, if we immunised animals with OVA-FITC, the DC that had captured the Ag were all Y-Ae^−^, proving the specificity of this mAb (Fig 4B). We next examined the dynamics of Ag-presenting DC in dLN after immunisation and found that they were similar in the presence or absence of CpG-B with a peak 48 h after immunisation (Fig 4C). DC express CD11c and MHCII molecules and have been categorised as CD8α^+^ and CD11b^+^-type DC, a dichotomy that takes into account phenotypic and functional attributes (Heath & Carbone, 2009; Guilliams *et al*, 2010). Ag-presenting DC in the dLN after immunisation were mainly CD11b^+^ CD8α^−^ (Fig 4D). Extensive phenotypic analyses of these latter showed that Ag-presenting CD11b^+^ DC could be divided into conventional DC (cDC) and monocyte-derived DC (moDC) based on CD64 expression (Fig 4E) (Langlet *et al*, 2012; Plantinga *et al*, 2013) with a majority of moDC at 48 h after immunisation (Fig 4F).

### CpG-B promotes IL-6 secretion by Ag-presenting moDC

**Figure 5:**
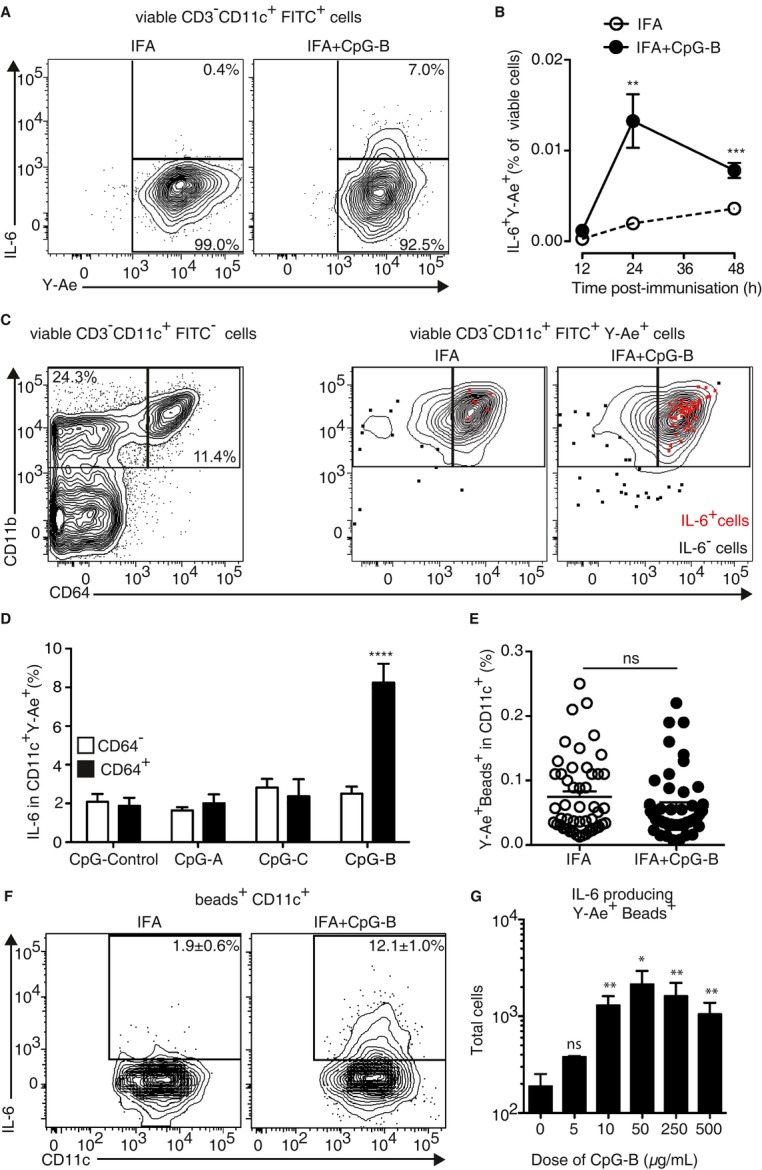
Ag-presenting moDC produce IL-6 in response to CpG-B. IL-6 production in CD11c^+^ FITC^+^ cells from dLN 2 days after s.c. immunisation with Ea-FITC in IFA or IFA+CpG-B. Kinetics of IL-6^+^CD11c^+^FITC^+^ cells in dLN after Ea-FITC s.c. immunisation with IFA or IFA+CpG-B (IFA, open circle; IFA+CpG-B, solid circle) (*n* ≥ 4/condition, mean ± s.e.m.). Expression of CD11b and CD64 at the surface of CD11c^+^FITC^−^, CD11c^+^FITC^+^IL6^−^ and IL-6^+^ cells in dLN 24 h after s.c. immunisation with Ea-FITC in IFA or IFA+CpG-B. Frequency of IL-6^+^ cells in CD11c^+^Y-Ae^+^ cells in dLN 2 days after s.c. immunisation with 10^10^ Ea-coated beads in IFA complemented with different type of CpG (*n* = 4/condition, mean ± s.e.m.). Total CD11c^+^ beads^+^ Y-Ae^+^ in dLN 2 days after s.c. immunisation with 10^10^ Ea-coated beads in IFA or IFA+CpG-B were analysed. IL-6 production was assessed in CD11c^+^ beads^+^ cells from dLN 2 days after s.c. immunisation with 10^10^ Ea-coated beads in IFA or IFA+CpG-B (*n* = 6/group, mean ± s.e.m.). Total cell count numbers of CD11c^+^ beads^+^ IL-6^+^ cells in dLN of mice 2 days after s.c. immunisation with 10^10^ Ea-coated beads in IFA or IFA complemented 1, 2, 10, 50 or 100 μg of CpG-B (*n* ≥ 4/group, mean ± s.e.m.) (G). Data information: Data are representative of at least three independent experiments. ns, nonsignificant; *P < 0.05; **P ≤ 0.01; ****P ≤ 0.001.

We explored the mechanism by which CpG-B-sensitised Ag-presenting DC could induce more Tfh cells *in vivo*. We performed intracellular stainings and found that Ag-presenting DC secreted much more IL-6 in animals immunised with IFA with CpG-B, 24 and 48 h after immunisation (Fig 5A and B). Strikingly, the majority of these cells were moDC as shown by CD64 expression (Fig 5C). As observed with the enhancing effect of CpG adjuvantation on Ag-specific Tfh cells and Ag-specific B cells, only type B CpG induced IL-6 secretion by Ag-presenting moDC (Fig 5D). We thus took advantage of the phagocytic capacity of moDC when compared to cDC and targeted them by using large beads (Randolph *et al*, 1999; Qu *et al*, 2004). Two days after immunisation with Ea-conjugated beads, we found that all the DC in the dLN that had captured the beads were as expected moDC only (Supplementary Fig S4). We also immunised animals with OVA-conjugated beads in IFA or IFA with CpG-B and observed that the total OVA-specific circulating IgG response day 14 after immunisation was enhanced after the addition of CpG-B (Supplementary Fig S5A). Strikingly, using 1W1K-conjugated beads, we observed that the addition of CpG-B to IFA increased 1W1K-specific Tfh-cell numbers in dLN 9 days after immunisation (Supplementary Fig S5B). Therefore, using large beads, we can target specifically moDC to present Ag in the draining LN that eventually prime an efficient T-cell-dependent B-cell response in response to CpG. We thus enumerated the number of moDC in dLN 2 days after immunisation with Ea beads and found no statistical difference between IFA and IFA with CpG-B-immunised animals (Fig 5E). Further, we found that the frequency of IL-6-producing moDC was increased in animals immunised with beads in IFA with CpG-B (Fig 5F). Noticeably, the number of IL-6-producing moDC was dependent on the dose of CpG-B added to IFA (Fig 5G). Together, these results demonstrate that CpG-B adjuvantation of other vaccine adjuvant enhances the frequency of IL-6-secreting Ag-presenting moDC and promote T-cell-dependent B-cell response.

### IL-6 production in response to CpG-B adjuvantation promotes Tfh-cell differentiation

**Figure 6:**
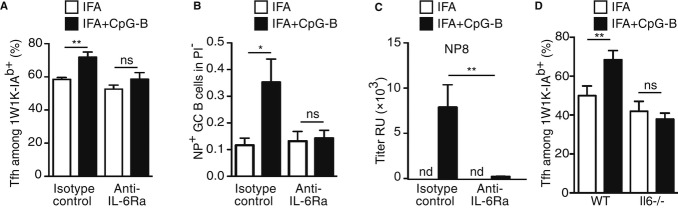
IL-6 produced by Ag-presenting DC in response to CpG-B adjuvantation promotes Tfh-cell differentiation. A Seven days after s.c. immunisation with 40 μg of 1W1K in IFA or IFA+CpG-B, dLN were collected and analysed for 1W1K-specific Tfh cells (1W1K-IA^b+^CD62L^lo^ CXCR5^+^) after treatment with anti-IL-6Rα mAb or isotype control (Rat IgG2b) (*n* = 5/group, mean ± s.e.m.). Frequency of Tfh among 1W1K-specific CD4^+^ T cells was estimated. B, C Twenty-one days after s.c. immunisation with 100 μg of NP-OVA in IFA or IFA+CpG-B, dLN were collected and analysed for NP^+^ GC-B cells (NP^+^CD138^−^B220^+^GL-7^+^CD95^+^) (B) and serum was collected for ELISA detection of NP8-specific IgG (C) (nd, not detected). D Eight weeks after reconstitution, chimeric CD11c-DTR (70%) + C57Bl/6 (30%) → C57Bl/6 (C57Bl/6) and CD11cDTR (70%) + IL-6^−/−^ (30%) → C57Bl/6 (IL-6^−/−^) mice were s.c. immunised with 1W1K in IFA or IFA+CpG-B. Frequency of Tfh among 1W1K-specific CD4^+^ T cells in dLN 9 days after s.c. immunisation from IFA-immunised chimeric mice treated three times with DTx (*n* = 4/group, mean ± s.e.m.). Data are representative of at least three independent experiments. ns, nonsignificant; *P <* *0.05; **P ≤* *0.01; ***P ≤* *0.005.

To explore whether highest production of IL-6 by CpG-B-sensitised Ag-presenting moDC plays a role in the enhancing effect induced by CpG-B, we next treated mice on days −1 and +4 (relative to immunisation) with a mAb that blocks IL-6 signalling *in vivo* (anti-IL-6Rα) and examined the activated CD4^+^ T cells in the dLN. As expected, in isotype control-treated animals, we observed that 1W1K-specific Tfh cells 7 days after immunisation were more numerous in animals immunised with IFA with CpG-B (Fig 6A). In contrast, this boosting effect was suppressed in anti-IL-6Rα-treated animals (Fig 6A). Moreover, we treated mice on days −1, +4, +9, +14, +19 with anti-IL-6Rα mAb and observed a smaller number of GC-B cells 21 days after NP-OVA immunisation in anti-IL6Ra mAb-treated animals than in isotype control-treated ones (Fig 6B). Interestingly, this observation correlated with a decrease in high-affinity NP8-specific Ig (Fig 6C). To directly document the role of IL-6 produced by DC, C57Bl/6 recipients were lethally irradiated before reconstitution with BM from CD11c-DTR and IL-6^−/−^ mice. The resulting chimeras were treated with DTx and immunised with 1W1K. We found that absence of IL-6 production in CD11c^+^ cells resulted in the absence of Tfh-cell differentiation enhancement due to CpG-B adjuvantation (Fig 6D). These results collectively demonstrate that the addition of CpG-B to other vaccine adjuvant directly increases the production of IL-6 by DC cells that, in turn, enhance Tfh-cell differentiation *in vivo*.

### moDC promote Tfh-cell responses

**Figure 7:**
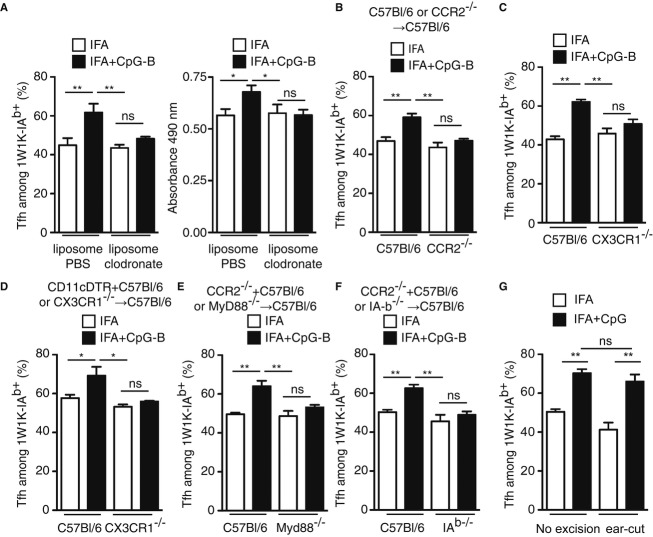
moDC drive the increase in Tfh-cell development due to CpG-B adjuvantation. A C57Bl/6 animals were s.c. immunised with 1W1K or OVA in IFA or IFA+CpG-B and treated twice with liposomes containing either PBS or clodronate. Nine days after s.c. immunisation with 1W1K, monocyte depletion was assessed in the blood of animals (data not shown) and the frequency of Tfh among 1W1K-specific CD4^+^ T cells was estimated in the corresponding animals (*n* = 5/group, mean ± s.e.m.). Fourteen days after s.c. immunisation with OVA, total OVA-specific IgG were estimated by ELISA (*n* = 5/group, mean ± s.e.m.). B–F Chimeric C57Bl/6 → C57Bl/6 (C57Bl/6) and CCR2^−/−^ → C57Bl/6 (CCR2^−/−^) mice 8 weeks after reconstitution (B), CXC3CR1 and wild-type littermates (C), chimeric CD11c-DTR (70%) + C57Bl/6 (30%) → C57Bl/6 (C57Bl/6) and CD11cDTR (70%) + CX3CR1^−^^/−^ (30%) → C57Bl/6 (CX3CR1^−^^/−^) mice treated three times with DTx (D), chimeric CCR2^−^^/−^ (70%) + C57Bl/6 (30%) → C57Bl/6 (C57Bl/6) and CCR2^−^^/−^ (70%) + MyD88^−/−^ (30%) → C57Bl/6 (MyD88^−^^/−^) mice (E) and chimeric CCR2^−^^/−^ (70%) + C57Bl/6 (30%) → C57Bl/6 (C57Bl/6) and CCR2^−^^/−^ (70%) + I-Ab^−^^/−^ (30%) → C57Bl/6 (I-Ab^−^^/−^) mice (F) were s.c. immunised with 1W1K in IFA or IFA+CpG-B. Nine days after s.c. immunisation, the frequency of Tfh among 1W1K-specific CD4^+^ T cells was estimated in the corresponding animals (*n* = 5/group, mean ± s.e.m.). G C57Bl/6 animals were immunised s.c. in the ear with 1W1K in IFA or IFA+CpG-B. 1 h after immunisation, ear was resected and the frequency of Tfh among 1W1K-specific CD4^+^ T cells was estimated in the corresponding animals 9 days after (*n* = 5/group, mean ± s.e.m.). Data information: Data are representative of at least three independent experiments. ns, nonsignificant; *P <* *0.05; **P ≤* *0.01.

We then turned our attention to test whether only moDC mediate the enhancing effect after CpG-B adjuvantation. First, we depleted *in vivo* phagocytic cells (monocytes and macrophages), but not cDC (presented in Supplementary Fig S6), using clodronate encapsulated in liposome. The resulting animals were immunised and we found that the 1W1K-specific Tfh-cell compartment and the OVA-specific IgG response were increased after CpG-B addition to other adjuvant only in PBS control-treated animals, but not in clodronate ones (Fig 7A). Moreover, in another series of experiment in which monocyte recruitment is impaired (CCR2^−/−^ chimeric mice, Fig 7B and CX3CR1^−/−^, Fig. 7C), we also found no increase in the Tfh compartment after IFA with CpG-B immunisation. One striking feature was that the 1W1K-specific Tfh compartment still developed and to the same extent in clodronate-treated animals or in CCR2^−/−^ chimeric mice and CX3CR1^−/−^ after IFA or IFA with CpG-B immunisation. These latter data suggested that cDC can prime the Tfh compartment in the absence of monocyte and moDC (Fig. 7A–C). In addition, we took advantage of mix BM chimeras (CD11c-DTR + CX3CR1^−/−^ into C57Bl/6) in which, after DTx treatment, there was only absence of moDC but presence of cDC that arose from the CXC3CR1^−/−^ BM and monocytes/macrophages that arose from the CD11c-DTR origin. In these animals, no enhancing effect on the Ag-specific Tfh-cell response was found 9 days after immunisation with 1W1K in IFA with CpG-B (Fig 7D). Finally, this enhancing effect was dependent of MyD88 signalling (Fig 7E) and I-A^b^ expression (Fig 7F) as shown in two different mix BM chimeras in which only CCR2^+^ cells were deficient for MyD88 or I-A^b^ expression. Strikingly, if 1 h after immunisation the site of immunisation was resected, CpG-B addition to the vaccine adjuvant had still an enhancing effect on Tfh-cell differentiation (Fig 7G). This latter experiment suggested that CpG-B and the Ag freely circulated to the dLN. Moreover, it also suggested that monocyte did not migrate to the site of immunisation but differentiated into moDC in the dLN. Altogether, this series of experiments demonstrated that cDC are implicated in the priming of CD4^+^ T cells and the differentiation of Tfh cells. However, moDC, in response to CpG-B, orchestrate additional Tfh-cell development by IL-6 secretion and Ag presentation, which eventually results in better B-cell protection.

## Discussion

In this study, we have collected evidence that CpG-B adjuvantation of other vaccine adjuvant strongly enhances Ag-specific T-cell-dependent B-cell responses by increasing the Tfh-cell compartment without changing the magnitude of the Ag-specific CD4^+^ T-cell pool. Somewhat surprisingly, the Tfh enhancing phenomenon was correlated with no difference in the nature of Ag-presenting DC or an increase in DC recruited to the dLN after immunisation. However, we have shown that CpG-B directly acts on moDC by inducing IL-6 production that, in turn, enhances Ag-specific Tfh-cell development and, consequently, GC-B-cell reaction and IgG response. We have documented this to be the case not only for IgG responses to soluble Ag emulsified in classical adjuvant (IFA) but also for two other adjuvants validated for clinical use, namely Alum and a MPL-based adjuvant.

The impact of TLR signalling on Ab responses remains controversial. Using chimeric mice that were deficient in MyD88 only in B cells, Pasare and Medzhitov have shown that Ag-specific Ab responses require the activation of B cells through TLR engagement (Pasare & Medzhitov, 2005). In contrast, using mice genetically deficient in TLR adaptors, Gavin and colleagues showed that TLR signalling was dispensable to induce a robust T-cell-dependent Ab response (Gavin *et al*, 2006). Very recently, Hou *et al* showed that soluble CpG had the ability to promote strong B-cell responses via DC and could therefore act as an adjuvant (Hou *et al*, 2011). As such, the most remarkable finding of our study is that adjuvantation with soluble CpG-B of other vaccine adjuvant can be used as an enhancer of Ag-specific T-cell-dependent Ab response without modifying the extent of the CD4^+^ T-cell pool. The enhancing effect of CpG-B is not restricted to one type of adjuvant but was observed for all three emulsions tested: aqueous (Alum), water-in-oil (IFA) and oil-in-water (squalene). The impact of CpG on Ab responses resulted from an increase in GC-B cells and circulating Ig but also from more GC with enlarged structures. This enlargement of GC could ultimately lead to more Ig somatic mutations and higher Ig affinity. However, we did not detect any significant increase in the binding of the total NP or OVA to Ag-specific B cells as monitored as the mean fluorescence intensity of NP or OVA staining and the increase in IgG response was observed irrespective of Ig affinity for the Ag. We also found that the increase in the total IgG response was not due to an increase in only one class of IgG but was spread to several IgG classes and was observed even at later time point after immunisation. This increase in the Ig response in the memory phase correlated with an increase in Ag-specific memory Tfh cells. While Tfh cells were considered as effector cells fully differentiated and prone to apoptosis until recently, we showed that protein vaccination selects locally effector Tfh cells that become memory Tfh cells and that remain in draining lymphoid tissue (Fazilleau *et al*, 2007). The unique capacity of these memory CD4^+^ T cells to specifically help the B cells after Ag rechallenge was demonstrated using reporter mice (Liu *et al*, 2012) and using transfer experiment of TG cells in wild-type animals (Weber *et al*, 2012), since circulating memory Tfh cells have also been identified in the human blood (Morita *et al*, 2011). Therefore, in our context, CpG-B does not only enhance the effector cells responsible for an efficient B-cell response but also promotes the formation of a larger pool of Ag-specific memory T and B lymphocytes. Altogether, these data clearly show that the addition of CpG-B to vaccine formulation enhances T-cell-dependent Ab response specific for the Ag, memory formation and long-term immunity.

One question raised by our observation was how CpG-B can induce a bias towards Tfh-cell differentiation. We were able to show that moDC through IL-6 secretion drive this phenomenon. In contrast, we also showed that in the absence of moDC *in vivo*, the priming of CD4^+^ T cells was still efficient and that some activated CD4^+^ T cells differentiated into Tfh cells. Furthermore, in the context of protein vaccination, it was previously shown that monocytes participate to Ag transport to the dLN (Calabro *et al*, 2011) and that vaccine adjuvants such as Alum or MF59, a squalene-based adjuvant, promote monocyte differentiation into moDC (Seubert *et al*, 2008). As such, our findings also parallel very recent studies showing in the lung that cDC are important in priming CD4^+^ T-cell response against allergens and that moDC can also present Ag to CD4^+^ T cells and coordinate inflammation locally (Plantinga *et al*, 2013). In this context, the authors showed that TLR4-dependent response induced more attraction of moDC to the lung, and some of these DC were then able to migrate to the dLN (Plantinga *et al*, 2013). After intramuscular injection, it was also shown that the addition of LPS to Alum increases the homing of moDC in the dLN (Langlet *et al*, 2012). To note, human homologs of moDC were identified, named inflammatory DC, and were also shown to orchestrate CD4^+^ T-cell responses (Segura *et al*, 2013). In our experimental settings, we observed that CpG-B favours IL-6 production by moDC to orientate CD4^+^ T cells towards the Tfh-cell lineage but has no impact on the attraction of moDC to the dLN since the number of Ag-presenting moDC in the different immunising conditions was similar. In contrast, by excising the site of immunisation, we were still able to observe the enhancing effect due to CpG-B adjuvantation, suggesting that monocyte differentiation into moDC might happen in the dLN where the resulting cells can respond to CpG stimulation, capture the Ag and present it to CD4^+^ T cells.

Earlier studies of Ag-specific CD4^+^ T cells in WT mice examined the contribution of TCR affinity and adjuvant in the CD4^+^ T-cell clonal selection process. It was demonstrated that only CD4^+^ T cells bearing TCR with an affinity for pMHCII above a threshold were selected (Malherbe *et al*, 2004). Subsequently, when CpG-B was used as an adjuvant, this was found to raise the selection threshold, which ultimately enhanced the overall TCR affinity of Ag-specific CD4^+^ T-cell repertoire (Malherbe *et al*, 2008). Using the same Ag model, we have shown that Tfh-cell differentiation is related to the strength of TCR binding and that Tfh cells predominantly express TCR with high affinity (Fazilleau *et al*, 2009b). In the present study, we found no detectable difference in the affinity of the whole 1W1K-specific CD4^+^ T-cell repertoire after IFA or IFA with CpG-B immunisation, as evaluated by the intensity of staining with fluorescent tetramers. This observation raises the question of how the proportion of Ag-specific Tfh cells can increase without a detectable change in the affinity of the selected TCR repertoire. One possibility is that the affinity threshold for Tfh-cell differentiation drops. In support of this hypothesis, Ag-presenting moDC harboured a similar activated phenotype but higher production of IL-6, a cytokine shown to be important for Tfh-cell differentiation *in vivo*. Consequently, more IL-6-producing Ag-presenting moDC mobilised in the dLN were enumerated after the addition of CpG-B. Elegant real-time studies performed *in vivo* have shown that competition for Ag and prolonged contact with DC control CD4^+^ T-cell clonal selection (Celli *et al*, 2007; Garcia *et al*, 2007). Therefore, an increase in IL-6-producing Ag-presenting DC could lower the affinity threshold for Tfh-cell differentiation without changing the overall affinity threshold for clonal selection of the whole Ag-specific CD4^+^ T-cell repertoire.

In summary, our results clearly indicate that both cDC and moDC participate in Tfh-cell differentiation. Anyhow, adjuvantation with soluble CpG-B of vaccine adjuvant enhances the adjuvant power of protein vaccine and increases the long-term protection through the promotion of the Ag-specific B-cell response. This phenomenon relies on the better differentiation of the key regulators of B cells, namely Tfh cells, and their increased differentiation by moDC. Because TLR9 agonist effects are focused on moDC and because human moDC express TLR9 and are sensible to CpG (Hoene *et al*, 2006), the addition of CpG opens new perspective for the formulation of adjuvant combinations.

## Materials and Methods

### Mice

C57Bl/6 mice were purchased from Centre d'Elevage Janvier. E. Barhaoui provided TLR9^−/−^, S. Fillatreau JHT, N. Pizzinat I-Ab^−/−^, H. Coppin IL-6^−/−^, S. Guerder CD11c-DTR and CD45.1, R. Burcelin Myd88^−/−^ and CX3CR1^−/−^ and T. Walzer CCR2^−/−^ mice. All mice were on the C57Bl/6 genetic background, and only females of 8–12 weeks of age were used for experimental procedures. All experiments were performed in accordance with national and European regulations and institutional guidelines, and mouse experimental protocols were approved by the local ethics committee (Regional approval No. 311155523, ethical review No. MP/19/58/06/12).

### Bone marrow chimeras

Mice were γ-irradiated (8.5 Gy (850 rad), ^137^Cs source) the day before i.v. injection of 1 × 10^7^ T-cell-depleted BM cells. BM cells were recovered from femur and tibia. After red cell lysis using Tris-buffered ammonium chloride, cells were enriched in lineage antigen-negative cells using the mouse Lineage Cell Depletion kit and LS/MS MACS columns (Miltenyi Biotec) according to the manufacturer instructions. Anesthetised recipients were intravenously injected through the retro-orbital sinus in 200 μl DMEM media. Transplanted mice were housed with antibiotics in their drinking water for 2 weeks and were analysed 8 weeks after reconstitution. In the case of restriction of genetic deficiencies to B cells and DC, a mix of 70% of BM from JHT and CD11c-DTR animals, respectively, was added to 30% of BM of the different deficient strains as described in figure legends. Chimeras with Cd11c-DTR BM were i.p. treated every second day with 4 ng/g body weight diphtheria toxin (Dtx) (Sigma-Aldrich) and 1 day before s.c. immunisation with 20 ng/mouse.

### Immunisation

Ovalbumin protein (OVA), Sigma Adjuvant System (SAS) and IFA were from Sigma-Aldrich, Ea (ASFEAQGALANIAVDKA), Ea-FITC, 1W1K (EAWGALANKAVDKA) from Genecust, Alum and OVA-Alexa488 from Invitrogen, NP-OVA from Biosearch Technologies Inc. CpG-A (5′-GGGGTCAACGTTGAGGGGGG-3′), CpG-B (5′-TCCATGACGTTCCTGACGTT-3), CpG-B control (5′-TCCATGAGCTTCCTGAGCTT-3′, same sequence that CpG-B but contains GpC dinucleotides instead of CpG and can therefore be used as a negative control for CpG-B), CpG-C (5′-TCGTCGTTTTCGGCGCGCGCCG-3′) were from Invivogen and carboxylated green fluorescent microspheres from Polysciences (Warrington, PA). Mice were either i.p. injected with 200 μl or immunised s.c. at the base of tail with 100 μl each side with 40 μg of peptide 1W 1K, 100 μg of OVA or NP-OVA, 200 μg of Ea-FITC or 1.2 × 10^10^ beads in the indicated adjuvant.

### *In vivo* treatment

i.p. injections of 100 μg D7715A7 mAb (anti-IL-6Rα, eBioscience) or rat IgG2b isotype control were performed at day −1 and +4 post-immunisation. i.v. injections of 250 μl of clodronate liposomes or control PBS liposomes (clodronateliposome.com) were performed from day −1 every second day.

### ELISA

ELISA plates (Thermo scientific) were coated with 10 μg/ml OVA. IgG were detected with horseradish peroxidase (HRP)-conjugated anti-mouse total IgG (Southern Biotech). The HRP substrate o-phenylenediamine dihydrochloride (OPD) was purchased from Sigma-Aldrich. IL-6 in sera was determined using IL-6 ELISA kit (eBioscience). NP-specific IgG (Southern Biotechnology Associates) was detected in plasma from blood by ELISA using the hapten NP conjugated to the protein carrier bovine serum albumin (BSA) for which the conjugation ratio was estimated. To detect high-affinity IgG, NP8 (8 NP conjugated to BSA) was used, NP15 for high- and intermediate-affinity IgG and all IgG affinities with NP25. All NP-BSA conjugates were from Biosearch Technologies Inc.

### Flow cytometry

For population analysis, draining lymph nodes (dLN, inguinal and periaortic after s.c. immunisation) and spleen (after i.p. injection) of immunised mice were removed. Cell suspensions were prepared in PBS/2% FCS, 5 mM EDTA. For DC analysis, organs were dissociated using 125 μg/ml LiberaseTL (Roche) and 40 μg/ml DNAase I (Sigma-Aldrich). For staining of Ag-specific cells, 1W1K-IA^b^ tetramer was obtained from NIH Tetramer core facility, NP-PE from Biosearch Technologies Inc. 1 × 10^8^ cells/ml cells were firstly stained at room temperature for 120 min with optimal concentration of tetramer or 60 min with NP-PE or OVA-FITC at a final concentration of 1 μg/ml. Then, cells were incubated on ice for 45 min with fluorophore (or biotin)-labelled mAbs. The following mAbs purchased from BD Biosciences were used: anti-Bcl-6, anti-CD11b, anti-CD138, anti-CD11b, anti-CXCR5, anti-CD3, anti-CD8α, anti-CD95. The following mAbs purchased from eBioscience were used: anti-B220, anti-CD4, anti-CD64, anti-Mar-1, anti-IL-6, anti-CD11c, anti-Ly-6C, anti-GL-7, anti-CD62L, anti-CD44, anti-F4/80, anti-IgD, biotin-Y-Ae. Anti-CCR2 was from R&D. For IL-6 intracellular staining, cell suspensions were incubated for 4 h at 37°C in the presence of 3 μg/ml Golgi Plug (BD Bioscience) and 4 μM monensin, fixed and permeabilised using BD Fixation/Permeabilisation kit. Before permeabilisation, cells were stained with Fixable Viability Dye eFluor450 or eFluor660 (eBioscience). Data were collected on a BD LSRII™ (BD Biosciences) and analysed using FlowJo software (Tree Star).

### Statistical analysis

Experiments were performed in mice at least three and up to nine times, amounting to between 6 and up to 25 individual samples. Differences between variables were evaluated using the nonparametric Mann–Whitney test. All statistical analyses were carried out with Prism 4.0 software (GraphPad). *P*-values < 0.05 were considered statistically different.

## The paper explained

### Problem

The formulation of effective vaccines is the central element of any public health armamentarium to induce protective immunity.

### Results

We found that adjuvantation of vaccine adjuvant such as Alum with CpG oligonucleotides, the TLR9 ligands validated for clinical use, promotes germinal centre reaction and enhances antibody response. This correlates with an increase in Tfh cells, while the overall magnitude of the antigen-specific CD4^+^ T-cell response is unchanged. We demonstrated that while conventional dendritic cells prime and initiate CD4^+^ T-cell responses, monocyte-derived dendritic cells orchestrate the Tfh cell-enhancing effect by activating CD4^+^ T cells towards the Tfh lineage.

### Impact

Targeting antigen presentation by monocyte-derived dendritic cells could enhance vaccine efficacy.
