# Supplementary material for: Monocyte-derived dendritic cells promote T follicular helper cell differentiation
Source: EMBO Mol Med. 2014 Apr 11;6(5):590–603. doi: 10.1002/emmm.201403841 (PMC4023883; doi:10.1002/emmm.201403841)
Supplement: Supplementary file 1 [file emmm0006-0590-sd1.pdf]

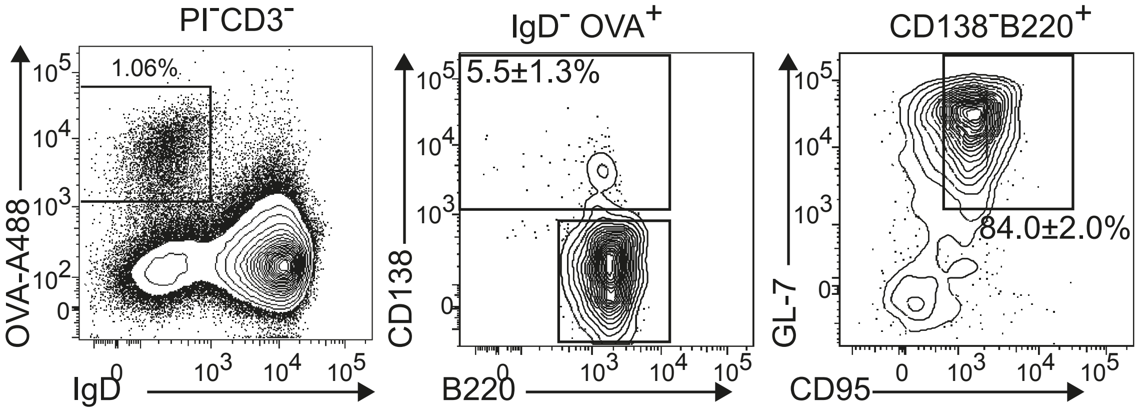

Figure S1:
Tracking OVA-specific B cells by Flow cytometry.
14 days after s.c. immunisation of C57Bl/6 mice with 100μg of OVA in IFA,
dLN (inguinal and periaortic) were collected and analysed to estimate total OVA-specific B cells.
Representative example of FACS profiles: OVA-specific B cells (OVA+ IgD-; left panel),
among which one can identify plasma cells (PC: CD138+; middle panel)
and GC B cells (B220+GL-7+CD95+; right panel).
Numbers adjacent to outlined areas indicate cell percentages (n=4, mean±SEM).
