# Supplementary material for: Monocyte-derived dendritic cells promote T follicular helper cell differentiation
Source: EMBO Mol Med. 2014 Apr 11;6(5):590–603. doi: 10.1002/emmm.201403841 (PMC4023883; doi:10.1002/emmm.201403841)
Supplement: Supplementary file 2 [file emmm0006-0590-sd2.pdf]

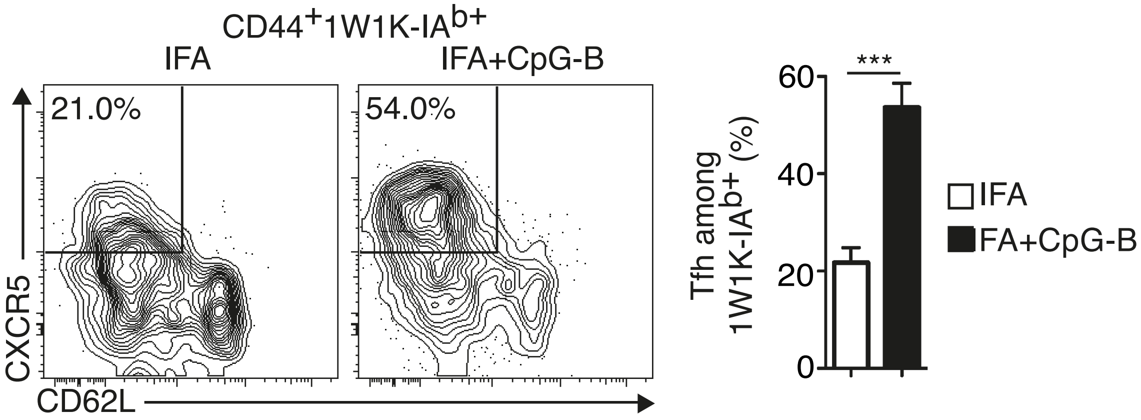

Figure S2:
Adjuvantation with CpG-B promotes memory Tfh cell development.
Frequency of 1W1K-specific memory Tfh cells in the dLN 60 days after immunisation of C57Bl/6 mice with 1W1K in IFA or IFA+CpG-B (n=5/group, mean±SEM).
Data are representative of three independent experiments.
*** $p \leq 0.005$
