# Supplementary material for: Monocyte-derived dendritic cells promote T follicular helper cell differentiation
Source: EMBO Mol Med. 2014 Apr 11;6(5):590–603. doi: 10.1002/emmm.201403841 (PMC4023883; doi:10.1002/emmm.201403841)
Supplement: Supplementary file 3 [file emmm0006-0590-sd3.pdf]

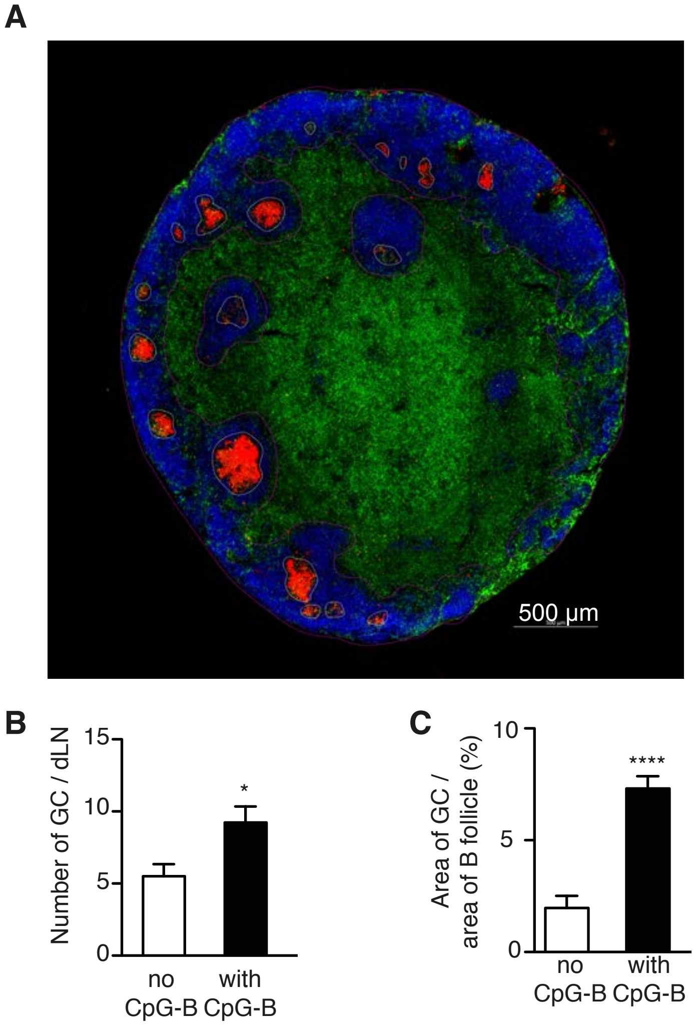

Figure S3:
CpG-B induces more GC in dLN
21days after immunisation with in IFA (no CpG-B) or IFA+CpG-B (with CpG-B), dLN were collected and sections were performed and stained with anti-IgD (blue), anti-CD4 (green) and anti-GL7 (red) (A). Number of GC (GL-7+) were enumerated (B) as well as surface area of GC out of the total surface area of B follicle (IgD+)(C) using the software Zen Lite (Carl Zeiss). (n=6/group, mean±SEM).
*$p \leq 0.05$; ****$p \leq 0.0001$
