# Supplementary material for: Monocyte-derived dendritic cells promote T follicular helper cell differentiation
Source: EMBO Mol Med. 2014 Apr 11;6(5):590–603. doi: 10.1002/emmm.201403841 (PMC4023883; doi:10.1002/emmm.201403841)
Supplement: Supplementary file 4 [file emmm0006-0590-sd4.pdf]

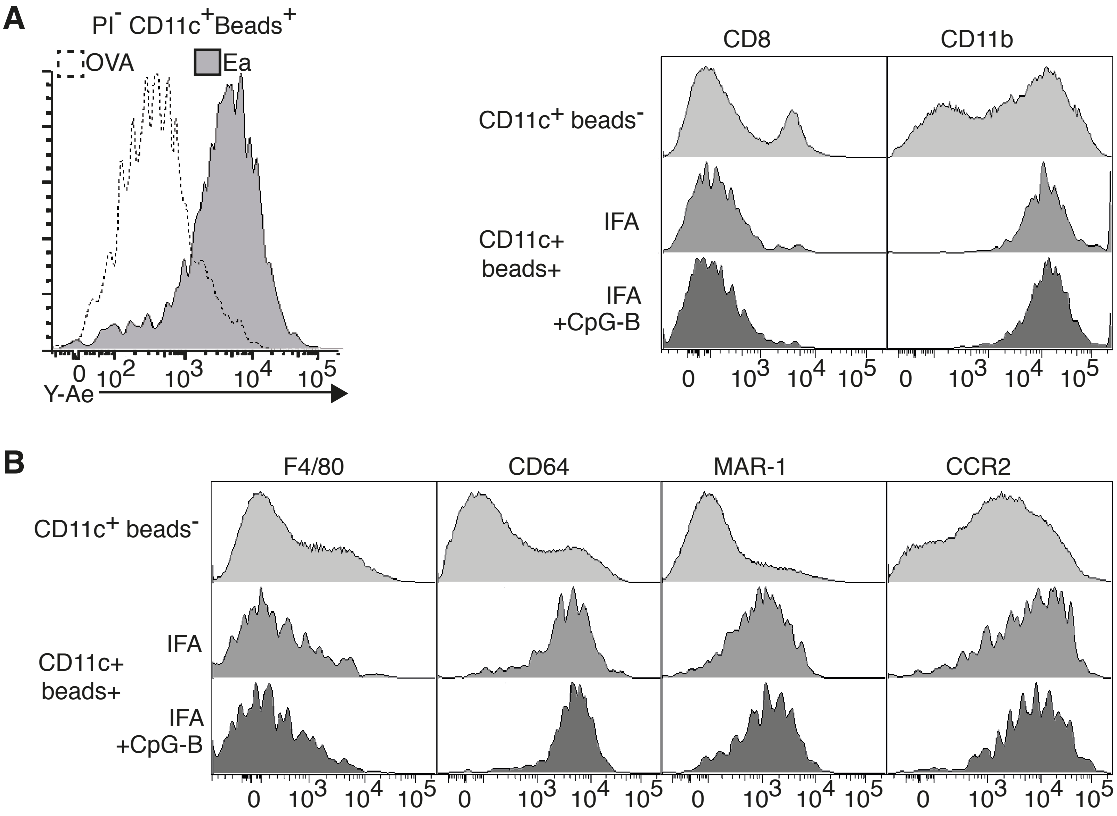

Figure S4 :

Ea-presenting DC after immunization with Ag-coated beads are moDC.

DC that captured and presented Ag ($CD11c^+$ $beads^+$ $Y\text{-}Ae^+$) in dLN of mice 2 days after immunisation with 10^{10} Ea-coated beads (filled histogram) emulsified in IFA were analysed.
Open histogram corresponds to mice immunised with 10^{10} beads coated with OVA in IFA (A).
2 days after immunisation with 10^{10} Ea-coated beads in IFA or IFA+CpG-B, dLN cells were analysed for CD11c expression and beads capture. $CD11c^+$ cells in dLN were also analysed for expressions of CD11b and CD8, F4/80, CD64, MAR-1 and CCR2 (B).
Data are representative of at least four independent experiments.
