# Supplementary material for: Monocyte-derived dendritic cells promote T follicular helper cell differentiation
Source: EMBO Mol Med. 2014 Apr 11;6(5):590–603. doi: 10.1002/emmm.201403841 (PMC4023883; doi:10.1002/emmm.201403841)
Supplement: Supplementary file 5 [file emmm0006-0590-sd5.pdf]

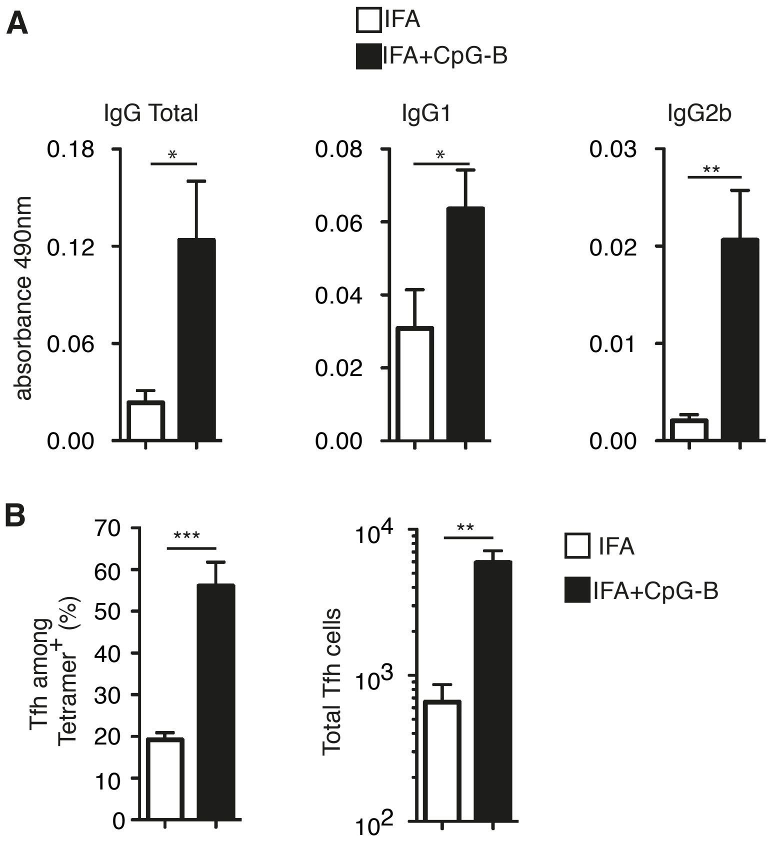

Figure S5:

Addition of CpG-B to Ag-conjugated beads increases Tfh-dependent B cell responses.

(A) OVA-specific total IgG, IgG1, IgG2b in sera of mice 14 days after immunisation with 10^{10} OVA-coated beads in IFA or IFA+CpG-B (n=5/group, mean±SEM).

(B) 9 days after immunisation with 10^{10} of 1W1K-coated beads in IFA or IFA+CpG-B, dLN were collected and analysed for 1W1K-specific Tfh cells (n=5/group, mean±SEM).

*p≤0.05; **p≤ 0.005; ***p≤0.0005
