# Supplementary material for: Monocyte-derived dendritic cells promote T follicular helper cell differentiation
Source: EMBO Mol Med. 2014 Apr 11;6(5):590–603. doi: 10.1002/emmm.201403841 (PMC4023883; doi:10.1002/emmm.201403841)
Supplement: Supplementary file 6 [file emmm0006-0590-sd6.pdf]

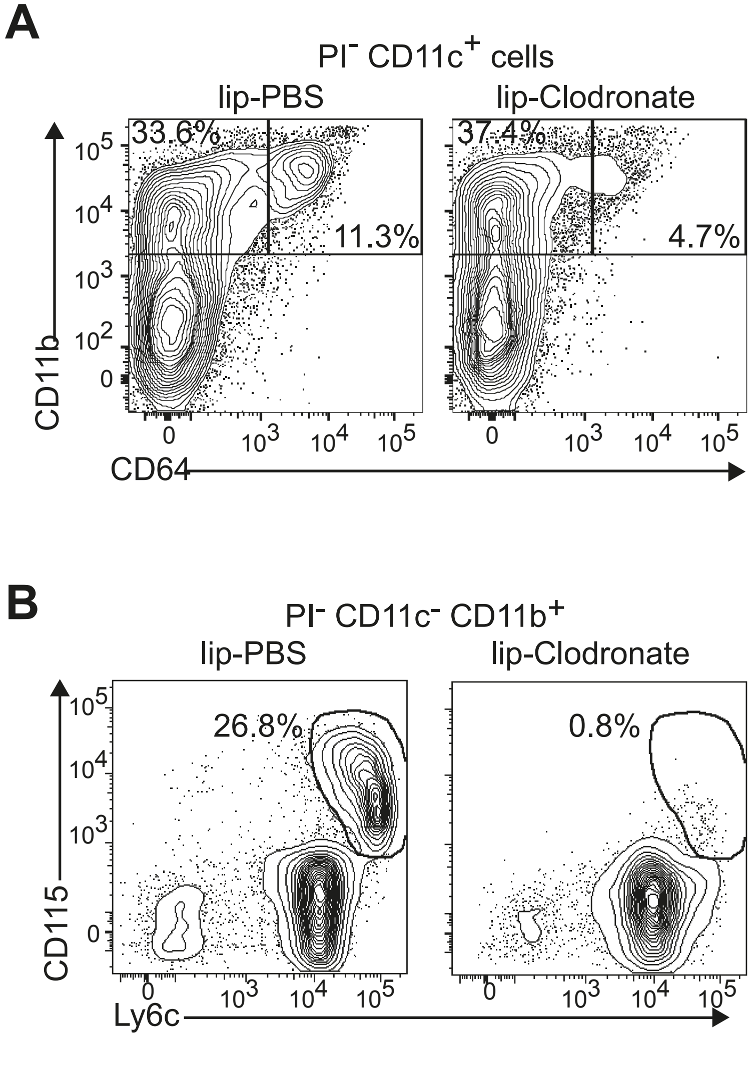

Figure S6: Impact of clodronate treatment
C57Bl6 animals were immunised with Ag in IFA with CpG and treated with liposomes containing either PBS or clodronate.
2 days after immunisation, the expression of CD11b and CD64 at the surface of CD11c+ cells in the draining LN was estimated (**A**) or 9 days after immunisation, monocyte depletion was assessed in the blood of animals (**B**)
(n≥4/group, mean ± SEM).
