# Supplementary material for: Monocyte-derived dendritic cells promote T follicular helper cell differentiation
Source: EMBO Mol Med. 2014 Apr 11;6(5):590–603. doi: 10.1002/emmm.201403841 (PMC4023883; doi:10.1002/emmm.201403841)
Supplement: Supplementary file 7 [file emmm0006-0590-sd7.pdf]

EMBO Molecular Medicine

Monocyte-derived Dendritic cells promote T Follicular Helper cell differentiation

Svetoslav Chakarov and Nicolas Fazilleau

Corresponding author: Nicolas Fazilleau, INSERM

Review timeline:

Submission date:	24 January 2014
Editorial Decision:	26 February 2014
Revision received:	07 March 2014
Editorial Decision:	11 March 2014
Accepted:	11 March 2014

Transaction Report:

(Note: With the exception of the correction of typographical or spelling errors that could be a source of ambiguity, letters and reports are not edited. The original formatting of letters and referee reports may not be reflected in this compilation.)

Editor: Céline Carret

1st Editorial Decision 26 February 2014

Thank you for the submission of your manuscript to EMBO Molecular Medicine. We have now received the enclosed reports from the three referees who were asked to assess it. As you will see the reviewers are globally supportive of publication pending minor revision.

The referees require better explanations and more details, especially in the Materials and Methods section. Referee 2 suggests improving the presentation of some figures. Finally, clarity should be improved when recommended.

In addition, we do have a few editorial comments that you may want to reply to at this stage should the manuscript be accepted:

-Please develop the section The Paper Explained and do consider that this section is meant for non-expert scientists. Please feel free to have aloof on our website for examples of TPE.

-Please provide the figures as individual figure files and in high resolution.

-In the M&M section, please complete the "mice" paragraph by indicating the age, gender, background of mice used. Please provide information about their housing too. Which regional and institutional rules do you refer to? Are these in accordance with the EU legislation? I would encourage you to refer to the ARRIVE guidelines for reporting our animal experiments (http://www.equator-network.org/reporting-guidelines/improving-bioscience-research-reporting-the-arrive-guidelines-for-reporting-animal-research/)

-Every paper now includes a 'Synopsis' to further enhance their discoverability. Synopses are displayed on the html and are freely accessible to all readers. They also include a short standfirst -

written by the editor - as well as 2-5 one-sentence bullet points that summarise the paper. These should be complementary to the abstract - i.e. not repeat the same text, and written by the authors. We encourage inclusion of key acronyms and quantitative information. Please use the passive voice. Could I ask you to provide the bullet points in a separate file/via email please?

Please submit your revised manuscript within 3 months. I look forward to seeing a revised form of your manuscript as soon as possible.

***** Reviewer's comments *****

Referee #1 (Comments on Novelty/Model System):

The study is based on well-designed experiments using very elegant in vivo models. The question of the study is of high interest as it is related to the mode of action of TLR agonists as vaccine adjuvant. The findings are novel, and well demonstrated by the data.

Referee #1 (Remarks):

This manuscript presents an elegant study of the mode of action behind the boosting effect of CpG oligonucleotides on antigen-specific humoral immunity in the context of immunization. The authors nicely and convincingly highlight the role of IL-6 production by monocyte-derived dendritic cells in leading to increase in Tfh differentiation. I only have minor comments, mostly related to the presentation of the data, and the description of the experimental procedures. The data are very convincing, and no new experiment is needed.

The Materials and Methods section needs to be markedly more detailed and precise. The reader should be able to repeat all the experiments described in the manuscript, and this is presently not possible. What is "Tea" from Charles River in the Mice section? The type of irradiator used must be described. Were mice pre-treated with antibiotics before/ after irradiation? The exact methodology used for making the chimera should be described. If the protocol refers to a previously published methodology, the original paper should be cited. How much CpG was used per mouse in microg? What was the volume of immunization? Were serial serum dilutions used for the determination of antibody titers by ELISA? The authors must describe precisely their protocol for staining NP-reactive and OVA-reactive B cells. Can the authors confirm that no re-stimulation was performed ex vivo before staining intracellular IL-6 in DC?

For each experiment, the authors must describe whether they immunized the mice s.c. or i.p., and precise which lymphoid organ was analyzed. If it is "dLN", they should describe which LN they used.

The authors refer to CD4+ T cells as Th cells. CD4+ T cells comprise cells that do not have classical helper function. I would suggest that the authors use the terminology "CD4+ T cells" instead of "Th cells" throughout the manuscript.

Figure 1: some error bars seem to be missing in graphs shown in panels G and H. In the figure legend, some panels seem to be inadequately quoted.

Figure 2: Is the staining shown in panel A intracellular or surface staining? Did the authors check that the cells they are studying are CD19+? The group of McHeizer-Williams previously published a paper on B220- memory B cells that turned out to be an artifact. It is important to make sure that the cells analyzed here do not include these B220- cells.

Figure 3: the authors should explicit precisely how the chimeras were made for restriction of genetic deficiencies to B cells and DC (How were BM mixed etc?), as mentioned above. Do the authors have data on TFH in complete TLR9 KO mice? If so, they should include them in the manuscript.

In panel C, the authors show normal formation of TFH cells in B cell-deficient mice. Some other groups reported that B cells were required for TFH formation. The authors should discuss this. Does it depend on the adjuvant used?

Figure 5: Panel A: Do the authors have data obtained using an isotype control Ab for the IL-6 intracellular cytokine staining?
Figure S2: How was the quantification of the germinal centre area performed?

Page 12 of the manuscript: It seems a word has been forgotten in the sentence "Moreover, we treated mice on days -1, +4, ..., and a smaller number of GC-B cells". Is a word missing between "a" and "smaller"?

Page 13: The authors label the pathway they describe as non-classical TFH development. It is not clear to me why this should be labeled as non-classical.

Discussion: The discussion would benefit from the integration of more general observations in the field of TFH development. The question of TFH memory cells is not discussed here. The authors should raise this question, and quote the work of Weber et al on this subject (EJI 2012, v 42 p1981). Also, IL-6 can have many other impacts on T cell responses. It is not clear here whether IL-6 acts directly on TFH cells, or promotes TFH indirectly. A striking example has been obtained in the context of helminth infection where it was shown that IL-6 can affect Treg phenotype (Smith et al. EJI 2014 v44 p150-61). This could be relevant here. Finally, other pathways of TFH development can be very important. For instance, TFH dramatically expand during Salmonella infection in MyD88 KO mice (Ko et al. EJI 2012 v42 p618-628). The authors should discuss these points and quote these studies.

Referee #2 (Comments on Novelty/Model System):

The work demonstrates that CpG has an effect on antigen specific Tfh cells, identifies for the first time the cellular/molecular mechanism at the basis of this activation pathway, and recognizes the IL-6 producing moDC as the key cellular population. The conclusions drawn by the authors are all well supported by the presented data, and have important consequences on the field of vaccine adjuvants and vaccination in general.

Referee #2 (Remarks):

The manuscript by Chakarov et al. shows that addition of the adjuvant CpG to other vaccine adjuvants is able to increase antigen specific T follicular helper cells in the mouse draining lymphonodes. It also describes for the first time that the effect on Tfh cells induction depends upon TLR9 engagement on moDC and production of IL-6 by these cells. Although it was previously shown that addition of CpG to an alum formulated vaccine is able to increase the frequency of total Tfh cells after immunization (Mastelic B. et al, J Immunol 2012; 189:5764-5772), the current work demonstrates that CpG has an effect on antigen specific Tfh cells. Despite this is only shown for a peptide antigen (1W1K peptide), the work presented identifies the cellular/molecular mechanism at the basis of this activation pathway, and recognizes the IL-6 producing moDC as the key cellular population. The conclusions drawn by the authors are all well supported by the presented data, and have important consequences on the field of vaccine adjuvants and vaccination in general. The work highlights the impact of moDC on the immune response and shows that these cells can be considered a target for vaccine adjuvants whenever an increase in antigen specific Tfh is required to support differentiation of long-lived plasma cells and memory B cells. Therefore this work is certainly suitable for publication in EMBO Molecular Medicine, however editing throughout the manuscript is required to improve clarity and readability especially to the benefit of scientists from fields other than immunology/vaccinology. In addition several points need to be specifically addressed by the authors.

Points to be addressed:
1) Page 3 lanes 10-12: "Upon recognition of CpG, the intra-cellular adapter molecule Myeloid differentiation factor 88(MyD88) induces phosphorylation of interferon regulatory factor 7 (IRF-7)": this is not precise, since MyD88 is not a kinases and does not phosphorylate IRF-7. This concept should be stated in a different way.

2) Page 4 last lane: the cited reference (Wack et al., 2008) shows that CpG induces an increase of Ab response at an early time point (2 weeks post second immunization), not a long-lasting Ab response. Modify text accordingly.

3) The term "CpG monotherapy" on pages 3 and 14 is misleading: the paper cited by the authors refers to the use of "soluble CpG" as adjuvant. Change text accordingly.

4) Measurements of the anti-NP antibodies with different affinity using NP8, NP15 and NP25, should be better explained from a methodological point of view, at least in the material and methods section.

5) Page 8 lanes 3-6: "Interestingly, we also found that increase of Ag-specific Tfh-dependent B cell responses after adjuvantation with CpG-B of vaccine formulation could be observed not only at the peak of the immune response but also 60 days after immunisation for the Ig response (Fig 2F) and for 1W1K-specific Tfh cells (Fig S1)." The sentence as written is not clear and should be reshaped, especially the last part.

6) Page 9 lane 5: the term "seric IL-6" should be changed to "serum IL-6".

7) Page 12: "Moreover, we treated mice on days -1, +4, +9, +14, +19 with anti-IL-6R_ mAb and a smaller number of GC-B cells 21 days after NP-OVA immunisation (Fig 6B)." This should be rephrased.

8) Figure 7: this is a critical figure in the manuscript since the data shown lead to the identification of the moDC as the key target population for the adjuvant effect of CpG. Therefore, the rationale for each of the experiments shown should be clearly stated so that also a less experienced reader may easily interpret the results. In particular, in panel B authors should explain why they used the CCR2-/-àB6 chimera and not directly the CCR2-/- mice, while in panel C the CX3CR1-/- mice were directly tested.

9) Page 14: the phrase "Somewhat surprisingly, the Tfh enhancing phenomenon was correlated with no difference in the nature and phenotype of Ag-presenting DC..." is not correct, since the authors show that CpG increase the number of IL-6 producing DC in dLN (Figure 5C). Correct text as needed.

10) Please add reference to Mastelic B. et al, J Immunol 2012; 189:5764-5772, as this work is key for the discussion of the data.

Points that could be addressed although not necessary required for publication:
1) Authors could show the effect of CpG addition on total, not antigen specific TfH cells, after immunization with OVA or NP-OVA, when they show an increase of OVA specific or NP specific GC B cells.

2) Authors could discuss if the CpG effect on the increase of antigen specific Tfh cells requires the presence of other adjuvants, or it is observed also with addition of CpG to a vaccine formulation in the absence of any other adjuvant.

Referee #3 (Remarks):

This is a beautiful study that convincingly demonstrates a role for TLR9 signaling in monocyte-derived DCs in enhancing Tfh differentiation and antibody responses via secretion of IL-6. The experiments are well performed and carefully controlled. I only have minor revisions to suggest and would recommend its acceptance.

Minor points
P6 line 7 Suggest two additions to make it easier for the reader (bold): after immunization with 1W1K..... Same line: At day 9, the peak of the effector response
p7- describe what control CpG-B is

p7 - serum instead of seric
Fig. 2D. Please show representative FACS blots to demonstrate you can convincingly identify OVA-specific GC B cells.
P10 much more IL-6 (instead of many more IL-6)
Fig. 5 - The authors have only looked for changes in IL-6. It would be interesting to know if IL-12 is also increased.
Fig. 7A. Clodronate is known to also deplete some DCs and other populations besides monocytes. It would be helpful to show Cd11c vs Cd11b plots before and after clodronate treatment, indicating the frequencies of the different known DC and macrophage populations.
P13. Explain better "one hour after immunization the site of immunization was cut" so that reader does not have to look through methods and legends to understand the ear was resected.
P13 monocytes

1st Revision - authors' response 07 March 2014

Response to Reviewers

We would like to thank the reviewers for their efforts regarding these studies and hope that the changes added to the revised manuscript meet with their approval. We were encouraged by the reviewers' interest and comments regarding the original manuscript and have modified our manuscript so, we believe, it now addresses their concerns and strengthens it. The scope and conclusions of the original manuscript remain largely unchanged but now many modifications have been added so the manuscript is more clear and precise. A point-by-point response to each reviewers' comments are below.

Point by point Issues of the Reviewers:
Reviewer 1:
In general, this reviewer was positive about the findings presented in the original manuscript. The first reviewer highlighted the novelty of the findings and stated that 'the data are very convincing, and no experiment is needed'.

Comments:
1) The Materials and Methods section needs to be markedly more detailed and precise. The reader should be able to repeat all the experiments described in the manuscript, and this is presently not possible.
Response: We agree that some details in the materials and methods section were missing and apologize for that. In the revised version of the manuscript we therefore clarified all the points raised by this reviewer.

2) *What is "Tea" from Charles River in the Mice section?*
Response: Transgenic TEa mice express a transgene encoding a TCRαβ specific for the pMHCII IAb-Ea52-68. In some supplemental experiments that we performed, we used this strain of mice. Nevertheless, in the original version of our manuscript as well as the revised version, none of these experiments were presented. We made the mistake not to remove it from the text. This has now been edited.

3) The type of irradiator used must be described. Were mice pre-treated with antibiotics before/after irradiation? The exact methodology used for making the chimera should be described. If the protocol refers to a previously published methodology, the original paper should be cited.
Response: All these statements have been added to the revised version of our manuscript in page 18.

4) How much CpG was used per mouse in microg? What was the volume of immunization?
Response: Depending on the experiment, the dose of CpG was different. For every experiment the concentration is stated. However, we forgot to precise what was the volume of immunisation that has now been added in the revised version of the manuscript page 19.

© EMBO

5) Were serial serum dilutions used for the determination of antibody titers by ELISA? The authors must describe precisely their protocol for staining NP-reactive and OVA-reactive B cells.
Response: Cells were stained with a final concentration of 1 mg/mL of OVA-FITC or 1 mg/mL of NP-PE for 1 hour at room temperature. This information has been added page 20.

6) Can the authors confirm that no re-stimulation was performed ex vivo before staining intracellular IL-6 in DC?
Response: We confirm that no re-stimulation was performed.

7) For each experiment, the authors must describe whether they immunized the mice s.c. or i.p., and precise which lymphoid organ was analyzed. If it is "dLN", they should describe which LN they used.
Response: We agree with this comment and have modified the text accordingly (pages 20, 32-36)

8) The authors refer to CD4+ T cells as Th cells. CD4+ T cells comprise cells that do not have classical helper function. I would suggest that the authors use the terminology "CD4+ T cells" instead of "Th cells" throughout the manuscript.
Response: We agree with this comment and have modified the text accordingly.

9) Figure 1: some error bars seem to be missing in graphs shown in panels G and H. In the figure legend, some panels seem to be inadequately quoted.
Response: It is true that SEM bars were not visible on the figure 1 due to the size of the dots that were masking them. We thus decreased dot size that now results in appearance of the SEM bars. Regarding the figure legend, it has also been modified page 32.

10) Figure 2: Is the staining shown in panel A intracellular or surface staining? Did the authors check that the cells they are studying are CD19+? The group of McHeizer-Williams previously published a paper on B220- memory B cells that turned out to be an artifact. It is important to make sure that the cells analyzed here do not include these B220- cells.
Response: Staining in panel A is a surface staining. As shown in the figure below, the anti-CD19 monoclonal antibody (clone 1D3) was used in some (but not all) experiments presented in Figure 2 and confirmed that the antigen-specific IgD^- $CD3^-$ cells were all $CD19^+$ confirming that they were B cells. Moreover, one other layer of evidence that our analysis included only B cells and no artifact relies on the dot plot analysis presented in Figure 2 panel A showing CD138 and B220 expression at the surface of NP-specific B cells with no cells that were $B220^-$ $CD138^-$.

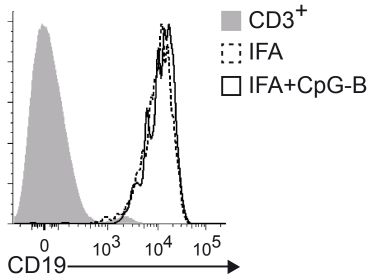

CD19 expression at the surface of dLN cells
14 days after s.c. immunisation with NP-OVA in IFA or IFA+CpG-B, dLN were collected and were analysed for the expression of CD19 at the surface of T cells (CD3+) or B cells (CD3- IgD- OVA+)

11) Figure 3: the authors should explicit precisely how the chimeras were made for restriction of genetic deficiencies to B cells and DC (How were BM mixed etc?), as mentioned above.
Response: We agree that some details were missing and again apologize for that. In the revised version of the manuscript we clarified how chimeras were made in the materials and methods section and in figure legends.

12) Do the authors have data on TFH in complete TLR9 KO mice? If so, they should include them in the manuscript.
Response: Unfortunately, we have no data on Tfh cells in $TLR9^{-/-}$ mice.

13) In panel C, the authors show normal formation of TFH cells in B cell-deficient mice. Some other

groups reported that B cells were required for TFH formation. The authors should discuss this. Does it depend on the adjuvant used?

Response: We agree that Tfh cell survival was shown to rely on the Tfh/B crosstalk and more precisely on ICOS/ICOS-L interaction. However, this is true for late timepoints (7 days after immunisation) during the course of an immune response, which is the reason why we studied Ag-specific Tfh cell formation in JHT mice early at day 5 after immunisation.

14) Figure 5: Panel A: Do the authors have data obtained using an isotype control Ab for the IL-6 intracellular cytokine staining?

Response: This is a fair criticism and an important control that we made the same time of the presented experiment. Anyhow, for a size purpose, we were not able to present this control nor in the original manuscript or in the modified version. This data are presented hereafter.

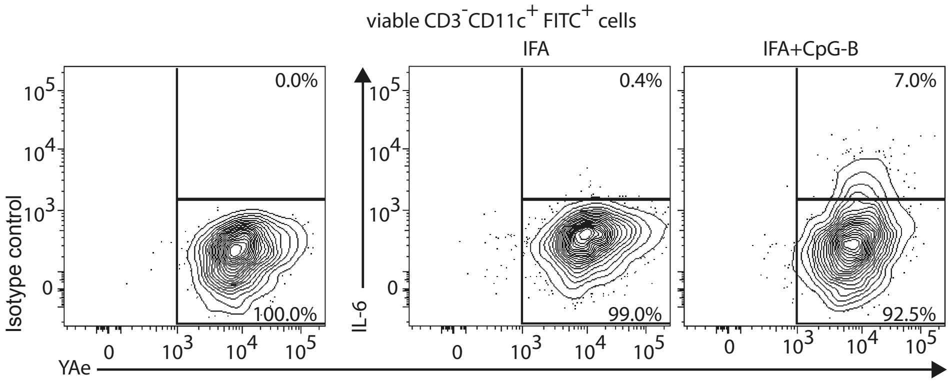

15) Figure S2: How was the quantification of the germinal centre area performed?

Response: This was done using the software Zen Lite (Carl Zeiss). The calculation made was: size area of GC / size area of B follicle. We modified the figure legend in Figure S3 accordingly.

16) Page 12 of the manuscript: It seems a word has been forgotten in the sentence "Moreover, we treated mice on days -1, +4, ..., and a smaller number of GC-B cells". Is a word missing between "a" and "smaller"?

Response: Indeed a word was missing and has been added to the revised version on page 12.

17) Page 13: The authors label the pathway they describe as non-classical TFH development. It is not clear to me why this should be labeled as non-classical.

Response: We believe that one of the findings of our study highlights that, in addition to a classical pathway of Tfh development, another one does exist. More precisely, the Tfh development due to cDC arises in absence or presence of CpG addition to vaccine adjuvant and leads to 40% of the Ag-specific T CD4+ cells to become Tfh cells. In response to CpG, moDC can also promote Tfh development in addition to the classical cDC-mediated pathway. Therefore, we agree that we made a mistake by using the term non-classical and removed it since it is only an additional pathway as stated page 13.

18) Discussion: The discussion would benefit from the integration of more general observations in the field of TFH development. The question of TFH memory cells is not discussed here. The authors should raise this question, and quote the work of Weber et al on this subject (EJI 2012, v 42 p1981). Also, IL-6 can have many other impacts on T cell responses. It is not clear here whether IL-6 acts directly on TFH cells, or promotes TFH indirectly. A striking example has been obtained in the context of helminth infection where it was shown that IL-6 can affect Treg phenotype (Smith et al. EJI 2014 v44 p150-61). This could be relevant here. Finally, other pathways of TFH development can be very important. For instance, TFH dramatically expand during Salmonella infection in MyD88 KO mice (Ko et al. EJI 2012 v42 p618-628). The authors should discuss these points and quote these studies.

Response: We have taken into account the reviewer comments and have modified the discussion. We, indeed, think that it is important to emphasize the potential implication of our observations at the memory level and not only in the effector phase. We thus modified the discussion accordingly in page 16. Moreover, we agree that the mode of action of IL-6 remains unknown in the context of our studies. However, it has been clearly demonstrated by many groups that the promotion of the Tfh lineage by IL-6 is direct, thus we believe it is not essential to discuss whether it is also true in our

experimental setting (Nurieva et al., Immunity 2008; PMID:18599325; Nurieva et al., Science, 2009, PMID:19628815; Suto et al., JEM, 2008, PMID:18474630; Dienz et al., JEM, 2009, PMID:19139170). Finally, we do not think that the data presented in the context of Salmonella infection in MyD88ko mice can be related to our studies since we clearly showed in figure 6 that IL-6 was the predominant molecule in the enhancing effect we observed. We therefore decided not to discuss this point as well.

Reviewer 2:
This reviewer was not concerned about the quality and relevance of the data that was presented since this reviewer thought this study was suitable for publication in EMBO Molecular Medicine. Anyhow, this reviewer was asking for editing of the manuscript so this latter would be strengthened and readable for a larger audience than immunologists only. We have attempted to allay all of these concerns in the rebuttal below. We believe these explanations will help to clarify the manuscript and hopefully satisfy the concerns of this reviewer.

Comments:
1) Page 3 lanes 10-12: "Upon recognition of CpG, the intra-cellular adapter molecule Myeloid differentiation factor 88(MyD88) induces phosphorylation of interferon regulatory factor 7 (IRF-7)": this is not precise, since MyD88 is not a kinases and does not phosphorylate IRF-7. This concept should be stated in a different way.
Response: We agree with this comment and have modified the text accordingly on page 3.

2) Page 4 last lane: the cited reference (Wack et al., 2008) shows that CpG induces an increase of Ab response at an early time point (2 weeks post second immunization), not a long-lasting Ab response. Modify text accordingly.
Response: We agree with this comment and have modified the text accordingly on page 4.

3) The term "CpG monotherapy" on pages 3 and 14 is misleading: the paper cited by the authors refers to the use of "soluble CpG" as adjuvant. Change text accordingly.
Response: We agree with this comment and have modified the text accordingly on pages 3 and 14.

4) Measurements of the anti-NP antibodies with different affinity using NP8, NP15 and NP25, should be better explained from a methodological point of view, at least in the material and methods section.
Response: We agree with this comment and have modified the text accordingly on page 21.

5) Page 8 lanes 3-6: "Interestingly, we also found that increase of Ag-specific Tfh-dependent B cell responses after adjuvantation with CpG-B of vaccine formulation could be observed not only at the peak of the immune response but also 60 days after immunisation for the Ig response (Fig 2F) and for 1W1K-specific Tfh cells (Fig S1)." The sentence as written is not clear and should be reshaped, especially the last part.
Response: We agree with this comment and have modified the text accordingly on page 8.

6) Page 9 lane 5: the term "seric IL-6" should be changed to "serum IL-6".
Response: We agree with this comment and have modified the text accordingly on page 9.

7) Page 12: "Moreover, we treated mice on days -1, +4, +9, +14, +19 with anti-IL-6R_ mAb and a smaller number of GC-B cells 21 days after NP-OVA immunisation (Fig 6B)." This should be rephrased.
Response: Indeed, a word was missing in the sentence and has been added to the revised version on page 12.

8) Figure 7: this is a critical figure in the manuscript since the data shown lead to the identification of the moDC as the key target population for the adjuvant effect of CpG. Therefore, the rationale for each of the experiments shown should be clearly stated so that also a less experienced reader may easily interpret the results. In particular, in panel B authors should explain why they used the CCR2-/-B6 chimera and not directly the CCR2-/- mice, while in panel C the CX3CR1-/- mice were directly tested.
Response: This is a fair comment and the rationale for each experiment was better explained in the revised version of the manuscript in page 13. Regarding the fact that we used BM chimera for

$CCR2^{-/-}$ and not directly the $CCR2^{-/-}$ animals was, unfortunately, not dictated by any scientific rationale. We studied both, CCR2 and CX3CR1 deficiencies, because it has been shown that they are differently expressed on monocyte population, patrolling versus inflammatory ones. It was therefore non redundant to study both deficiencies. We did BM chimera for the CCR2 experiment because the $CCR2^{-/-}$ animals we obtained from our collaborators had a health report that did not allow us to house them in our animal facility. Because CCR2 is expressed only on hematopoietic cells, we therefore decided to perform BM chimera since this was possible in our animal facility. We know it is not satisfying scientifically, but we strongly believed that the results obtained in the $CCR2^{-/-}$ animals would have been similar to the ones observed in BM chimera and consequently, the conclusions would have been the same.

9) Page 14: the phrase "Somewhat surprisingly, the Tfh enhancing phenomenon was correlated with no difference in the nature and phenotype of Ag-presenting DC..." is not correct, since the authors show that CpG increase the number of IL-6 producing DC in dLN (Figure 5C). Correct text as needed.
Response: Indeed, IL-6 production is a phenotypic feature so the sentence was modified on page 14.

10) Please add reference to Mastelic B. et al, J Immunol 2012; 189:5764-5772, as this work is key for the discussion of the data.
Response: As also asked by Reviewer#1 point 18, the paper by Mastelic et al has now been discussed in page 15.

Points that could be addressed although not necessary required for publication:
1) Authors could show the effect of CpG addition on total, not antigen specific TfH cells, after immunization with OVA or NP-OVA, when they show an increase of OVA specific or NP specific GC B cells.
Response: We also observed an increase of the pool of Tfh cells after immunisation with CpG without modifying the pool of activated $CD44^{hi}$ $CD4^{+}$ T cells. Anyhow, this effect is local (in the draining LN) and cannot be found systemically suggesting that the increase seen of the Tfh pool actually mainly reflects the increase of the antigen-specific Tfh cells. We therefore believed that it is less convincing to present this set of data than the ones using pMHCII tetramers.

2) Authors could discuss if the CpG effect on the increase of antigen specific Tfh cells requires the presence of other adjuvants, or it is observed also with addition of CpG to a vaccine formulation in the absence of any other adjuvant.
Response: Unfortunately it is difficult to answer to this question since the fair comparison would be to monitor the antigen-specific Tfh cell responses after soluble CpG immunisation compared to PBS only-immunised animals. Anyhow, immunisation using no adjuvant leads to tolerizing conditions with almost no activation of antigen-specific CD4+ T cells. Again we have shown that CpG addition promotes IL-6 production of moDC that, in turn, promote Tfh cell development. In the absence of other adjuvant, we therefore think that CpG would act only on cDC since almost no moDC would be present. In conclusion, even with no experimental evidence, we believe that the enhancing effect due to CpG that we observed relies on an inflammatory context and therefore on combination of CpG to another vaccine adjuvant.

Reviewer 3:
We would like to thank this reviewer for encouraging comments about the novelty, quality and control of the work and appreciate the value of their constructive critique. In response to these comments, we have now modified our manuscript as described below.

Comments:
1) P6 line 7 Suggest two additions to make it easier for the reader (bold): after immunization with 1W1K..... Same line: At day 9, the peak of the effector response
Response: We agree with this comment and have modified the text accordingly on page 6.

2) p7- describe what control CpG-B is
Response: We have clarified in the materials and methods section what is the control CpG-B on page 19.

3) p7 - serum instead of seric

Response: We agree with this comment and have modified the text accordingly on page 7.
4) Fig. 2D. Please show representative FACS blots to demonstrate you can convincingly identify OVA-specific GC B cells.
Response: It is true that we did not show any FACS dot plots of OVA-specific B cells in the original manuscript. In order to answer to this reviewer comment, we have added a supplementary figure with representative FACS analysis as supplementary figure 1 (Fig S1) and have modified the text accordingly in page 7.

5) P10 much more IL-6 (instead of many more IL-6)
Response: We agree with this comment and have modified the text accordingly on page 10.

6) Fig. 5 - The authors have only looked for changes in IL-6. It would be interesting to know if IL-12 is also increased.
Response: It is true that it would be of interest to test whether CpG addition leads to more changes in the phenotype of moDC and/or cDC. Anyhow, we focused our attention on the IL-6 production by moDC in response to CpG and did not test whether IL-12 is also increased.

7) Fig. 7A. Clodronate is known to also deplete some DCs and other populations besides monocytes. It would be helpful to show Cd11c vs Cd11b plots before and after clodronate treatment, indicating the frequencies of the different known DC and macrophage populations.
Response: It is true that clodronate is not specific of monocyte depletion only and has been shown to have an effect on some DC and macrophage populations. Anyhow, in the context of i.v. injection, we investigated the effect of clodronate on $CD11c^+$ DC of the draining LN 48 hours after immunisation. We found that only moDC ($CD11b^+$ $CD64^+$) were depleted while nor $CD11b^+$ cDC or $CD8a^+$ cDC ($CD11b^-$ here) frequencies were impacted by clodronate treatment as compared to control-treated animals. Moreover, the impact of clodronate treatment in the blood was also evaluated and showed a total disappearance of $CD11b^+$ $CD115^+$ $Ly6C^{hi}$ cells. These informations have now been added as a supplementary figure (Fig S6) and described in the text page 13.

8) P13. Explain better "one hour after immunization the site of immunization was cut" so that reader does not have to look through methods and legends to understand the ear was resected.
Response: Indeed, we have modified the text in pages 13 and 37.

9) P13 monocytes
Response: Sorry but we do not understand this reviewer comment.

2nd Editorial Decision 11 March 2014

We are pleased to inform you that your manuscript is accepted for publication and is soon being sent to our publisher to be included in the next available issue of EMBO Molecular Medicine.
Congratulations on your interesting work.

© EMBO
